# Biomolecular condensates: molecular structure, biological functions, diseases, and therapeutic targets

**DOI:** 10.1186/s43556-025-00350-y

**Published:** 2025-11-05

**Authors:** Sunkyung Choi, Jung-Min Lee, Kee K. Kim

**Affiliations:** 1 Department of Biological Sciences, College of Natural Sciences, Keimyung University, Daegu, 42601 Republic of Korea; 2 Department of Biochemistry, College of Natural Sciences, Chungnam National University, Daejeon, 34134 Republic of Korea

**Keywords:** Phase separation, Biomolecular condensate, Nuclear speckle, Paraspeckle, Stress granule

## Abstract

Cells constantly encounter environmental and physiological fluctuations that challenge homeostasis and threaten viability. In response to these cues, specific proteins and nucleic acids engage in multivalent interactions and undergo phase separation to form membraneless assemblies known as biomolecular condensates. Nuclear condensates include paraspeckles, nuclear speckles, and Cajal bodies, while cytoplasmic condensates include stress granules, processing bodies, RNA transport granules, U-bodies, and Balbiani bodies. These assemblies regulate transcription, splicing fidelity, RNA stability, translational reprogramming, and integration of signaling pathways, thereby serving as dynamic platforms for metabolic regulation and physiological adaptation. However, dysregulation of these condensates has been increasingly recognized as a central pathogenic mechanism in neurodegenerative diseases, cancers, and viral infections, contributing to toxic protein aggregation, nucleic acid dysregulation, and aberrant cell survival signaling. This review provides a comprehensive synthesis of the molecular mechanisms governing condensation, delineates the diverse types and functions of major biomolecular condensates, and examines therapeutic approaches based on their pathophysiological relevance to disease development and progression. Furthermore, we highlight the cutting-edge technologies, including CRISPR/Cas-based imaging, optogenetic manipulation, and AI-driven phase separation prediction tools, which enable the real-time monitoring and precision targeting of cytoplasmic biomolecular condensates. These insights underscore the emerging potential of biomolecular condensates as both biomarkers and therapeutic targets, paving the way for precision medicine approaches in condensate-associated diseases.

## Introduction

Cells maintain life by continuously rebalancing molecular reactions in the face of fluctuating environments and intrinsic cues. Cells achieve rapid and reversible control over complex reaction networks without relying solely on membrane-bound organelles [1]. This important mechanism, first observed in the 1830s, occurs through the formation of biomolecular condensates, commonly called membraneless organelles [2]. Unlike classical membrane-bound organelles, biomolecular condensates dynamically assemble and disassemble in response to stimuli, thereby enabling rapid adaptation [3, 4]. These condensates compartmentalize specific proteins and nucleic acids, effectively rewiring the intracellular environment. Proteins and nucleic acids can self-organize through multivalent interactions and phase separation to form biomolecular condensates [5, 6]. In recent years, there has been increasing focus on how these processes are spatially and temporally organized within cells via the formation of biomolecular condensates. These condensates create selective, dynamic microenvironments that concentrate enzymes, substrates, and cofactors, buffer noise in signaling networks, and orchestrate information flow from transcription to translation.

Biomolecular condensates can be broadly classified into nuclear and cytoplasmic types, each with distinct functional profiles. Nuclear condensates, including nuclear speckle, paraspeckle, and Cajal body, facilitate the reorganization of transcriptional and post-transcriptional regulatory processes [7–9]. In parallel, cytoplasmic condensates such as stress granule, processing body (P-body), RNA transport granules, U-body, and Balbiani body (B-body) mediate translational arrest, mRNA storage and decay, and signal transduction regulation [10–14].

Importantly, failure to properly regulate biomolecular condensates has emerged as a unifying feature in the pathogenesis of several diseases [15]. Dysregulated phase separation results in persistent or aberrant condensation, which interferes with nucleic acid metabolism and protein homeostasis [16]. In neurodegenerative disorders such as amyotrophic lateral sclerosis (ALS) and frontotemporal dementia (FTD), the pathogenic solidification of condensates impairs neuronal resilience [17, 18]. In cancer cells, altered condensate dynamics may promote stress tolerance, apoptotic resistance, and immune evasion [19, 20]. During viral infections, host condensates can be subverted to facilitate viral replication or block antiviral responses [21]. These observations underscore the importance of dissecting condensate behavior, specifically in certain contexts where protective functions can be hijacked and transformed into pathological mechanisms. Consequently, the therapeutic modulation of condensates represents a promising frontier. These approaches open new avenues for intervention in disease-relevant condensate transformations without disrupting normal cellular functions [22].

This review explores the molecular structures and mechanisms by which biomolecular condensates form and function, their context-dependent roles across subcellular compartments, and their potential as guardians of cellular integrity and contributors to diseases. By highlighting recent insights and emerging technologies, we underscore why understanding condensates is essential for advancing both basic science and translational applications in precision medicine.

## Molecular structure of biomolecular condensates formation

Certain proteins form dynamic intracellular condensates through a reversible biophysical phenomenon known as phase separation [23, 24]. Phase separation refers to the spontaneous demixing of a homogeneous solution containing multiple molecular components into two distinct phases, resulting in the formation of biomolecular condensates [25–27]. One of these phases is enriched with specific macromolecules, whereas the other is a dilute phase, from which these components are relatively depleted [26]. The dense-to-dilute partitioning varies depending on the concentration of the components and the degree of interaction with their partner molecules [28]. Unlike classical membrane-bound organelles, which are encapsulated by lipid bilayers and function in segregated compartments, biomolecular condensates are membraneless and undergo dynamic molecular interactions, exchanging constituents with the surrounding environment. Despite the lack of a bounding membrane, these structures are stably maintained in eukaryotic cells, where their architectural integrity and biological functions are preserved [27]. Multiple mechanisms can contribute to condensate assembly in cells, including scaffolded assembly, bridging-induced clustering on polymer substrates such as chromatin, polyphase complex coacervation driven by electrostatic association, and percolation or gelation that forms viscoelastic networks, which can operate alone or together with liquid–liquid phase separation [6, 29, 30]. Consequently, phase separation is regarded as a central mechanism governing the formation and regulation of membraneless organelles [26, 31].

**Fig. 1:**
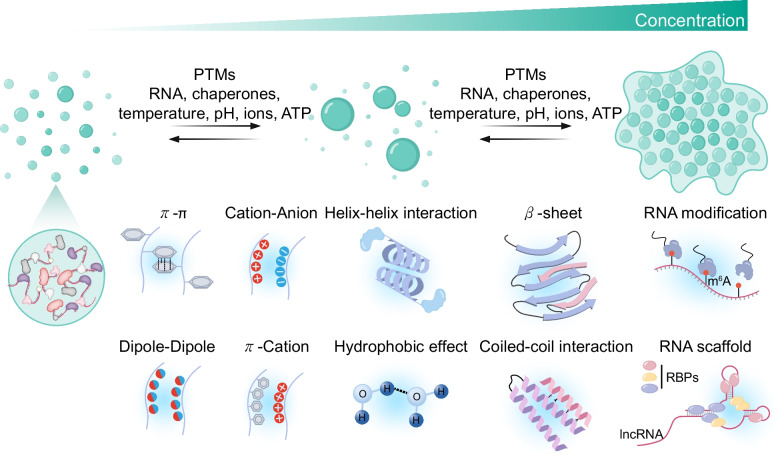
Mechanisms of biomolecular condensation via phase separation. Intrinsically disordered regions (IDRs) in proteins and RNA scaffolds establish multivalent contacts (π–π, electrostatic, π–cation, and hydrophobic interactions), driving condensate nucleation and subsequent protein–RNA recruitment. Post-translational modifications (PTMs) further modulate assembly or disassembly by altering interaction surfaces. RNA serves as both scaffold and client, with its structure, length, and modifications shaping condensate dynamics. Temperature, pH, ionic strength, and crowding conditions also determine phase separation thresholds

The primary driving forces of phase separation are multivalent interactions (Fig. 1) [31, 32]. These interactions often occur between proteins and nucleic acids or among nucleic acids themselves, facilitating local enrichment of biomolecules. Examples of such interactions include π–π stacking, cation–π interactions, electrostatic interactions between cations and anions, dipole–dipole interactions, and hydrophobic effects [33–41]. In the context of RNA-binding proteins (RBPs), cation–π interactions involving arginine–glycine–glycine (RGG/RG) motifs have been identified as key modulators of phase separation [42, 43]. Additionally, structural motifs such as helix–helix interactions, β-sheets, coiled-coils, steric zippers, and oligomerization domains also contribute to phase separation [44]. High-affinity multivalent interactions with defined binding partners markedly lower the threshold for condensation and stabilize assemblies [45]. In stress granule assembly, for instance, G3BP1 induces an RNA-dependent phase separation in response to increased intracellular free RNA concentration, illustrating the central role of protein–RNA multivalency [46]. At the molecular level, multivalent interactions in proteins are often mediated by intrinsically disordered regions (IDRs) or low-complexity domains (LCDs) [20, 47]. Some of these regions lack hydrophobic residues, rendering them incapable of autonomously folding into stable tertiary structures [31, 33, 48, 49]. Instead, they adopt extended, conformationally flexible states that allow access to a broad structural space, thereby providing multiple interaction sites that facilitate multivalent binding events. Conversely, the presence or substitution of hydrophobic residues within the motif can enhance phase separation by altering binding affinity with partner molecules or forming hydrophobic cores [50, 51]. This feature has been reported to be particularly important in disease-causing proteins, such as TDP-43 and alpha-synuclein, which are associated with neurodegenerative diseases [52, 53]. Thus, IDRs possess conformational biases that depend on their amino acid sequence [54]. These conformational biases may be induced by multivalent interactions that induce attraction or repulsion between distal regions of the IDR. These interactions initiate the nucleation of condensates, which subsequently grow through the recruitment of additional molecules from the surrounding environment.

Nucleation and growth of protein condensates are highly sensitive to various physicochemical perturbations, including temperature, pressure, pH, osmolarity, ionic strength, hydrogen bonding dynamics, reactive oxygen species, light exposure, molecular crowding, and fluctuations in the concentration or complexity of specific biomolecules [55–58]. Post-translational modifications (PTMs) of proteins also play crucial roles in modulating condensate dynamics. Phosphorylation of serine, threonine, or tyrosine residues; methylation; citrullination; ADP-ribosylation; acetylation; SUMOylation; and ubiquitination are known to alter interaction surfaces and modulate the affinity of intermolecular contacts [59, 60]. For example, phosphorylation introduces negatively charged phosphate groups that can disrupt electrostatic balance, thereby promoting or inhibiting condensate formation. Recently, phosphorylation within the IDRs of SRRM2 and Ki-67 has been reported to enhance molecular interactions within the network, thereby regulating the material properties of condensates by generating alternating charge blocks, thus increasing the propensity for phase separation [61, 62]. Conversely, mitotic hyperphosphorylation of NPM1 reduces charge blocks and inhibits phase separation, leading to nucleolar dissolution [62]. The addition of negatively charged groups can enhance or diminish this charge blockiness of the IDRs, potentially influencing condensate formation. Similarly, ubiquitination may alter protein stability and affect condensation and disassembly. Asymmetric dimethylation of arginine attenuates the driving forces for phase separation, thereby increasing the turnover dynamics of biomolecular condensates [33, 63].

The key molecular components that initiate condensate nucleation are referred to as scaffolds, whereas other co-phase-separating molecules are typically termed clients [3, 64]. Nucleic acids frequently act as molecular scaffolds, recruiting proteins to initiate phase separation. Through electrostatic interactions and repetitive base pairing, nucleic acids can serve as structural scaffolds or nucleation cores, thereby stabilizing condensates and determining their physicochemical properties. Nuclear long noncoding RNAs (lncRNAs), such as *NEAT1* and *MALAT1*, have been shown to engage in multifaceted interactions not only with RBPs but also with other RNAs and chromatin [65–67]. These RNAs are critical for layered assembly within condensates and may mediate intercondensate tethering, suggesting a structural role for RNA in spatial positioning and functional compartmentalization.

The effect of RNA on phase separation is influenced by multiple factors, including sequence, length, chemical modifications, and secondary structure. Among these, N^6^-methyladenosine (m^6^A), the most prevalent internal modification in eukaryotic mRNA, modulates RBP-binding affinity and RNA structure, ultimately affecting both the formation and maintenance of condensates [68, 69]. These findings support the notion that RNA modifications function as regulatory switches integrating environmental signals into intracellular responses. Alterations in protein and nucleic acid properties in response to internal or external cues can shift the critical concentration threshold for phase separation, thereby significantly influencing the formation or dissolution of condensates [70, 71]. This enables cells to rapidly reorganize their condensate composition within seconds to minutes in response to environmental changes, supporting the dynamic regulation and adaptive control of biomolecular assemblies. Such responsiveness is a hallmark of cellular stress responses and fundamentally contributes to the maintenance of cellular homeostasis.

Beyond structural roles, the physicochemical properties of condensates are intimately linked to their biological functions [3, 23]. Due to their high local macromolecular density relative to the surrounding cytoplasm or nucleoplasm, condensates act as microenvironments for spatial organization within the cell. Biomolecular condensates often serve as hubs where nucleic acids and proteins are highly concentrated, leading to reduced molecular diffusion and enhanced reaction kinetics [26, 31, 72]. Conversely, the sequestration of specific components within condensates can repress certain biochemical reactions [73, 74]. For example, stress granules can sequester RACK1, thereby suppressing apoptosis [75]. Through such mechanisms, RBP condensates regulate essential aspects of RNA metabolism, including mRNA stability, splicing, degradation, transport, and translation, by locally concentrating processing factors and translational machinery [27, 76–79]. Moreover, in response to cellular stress, condensates dynamically reorganize their composition, exerting spatiotemporal control over a wide range of processes, such as chromatin architecture, genome stability, DNA damage response and repair, transcription, and signaling pathways [80]. Notably, numerous lncRNAs localize to chromatin and form RNA "clouds" in specific nuclear compartments, where they exert gene regulatory functions. Thus, RBP condensates represent a key adaptive survival mechanism that enables cells to efficiently manage stress responses and maintain their viability.

Condensates transition from a droplet state to gels or soft glasses, maturing over time, as demonstrated by fluorescence recovery after photobleaching (FRAP), GFP fluorescence recovery, coalescence, and active and passive microrheology experiments [18, 81–84]. Mature condensates exhibit reduced fusion propensity and longer recovery times after photobleaching, indicating a significant reduction in internal molecular diffusion [1, 53, 85]. It has become clear that the material properties of condensates far exceed those of liquids. Although condensates in the droplet state allow rapid molecular exchange, prolonged persistence can lead to reduced dynamics, resulting in a transition to liquid-, gel-, or even solid-like states [18, 34, 86–89]. It has been reported that rigidification is promoted by increasing the binding affinity with partner molecules and inhibiting condensate disassembly through changes in temperature or salt concentration, PTMs, protein mutations such as those in FUS, and protein folding and misfolding events [18, 82, 90–92]. These transitions have been implicated in the pathogenesis and progression of various diseases. Therefore, a comprehensive understanding of the complex and dynamic mechanisms underlying the effects of biomolecular condensates will offer potential avenues for therapeutic intervention in condensate-related diseases.

## Compositions and properties of biomolecular condensates

Biomolecular condensates arise in response to developmental, metabolic, and stress cues through phase separation that concentrate selected proteins and nucleic acids into dynamic compartments. Nuclear and cytoplasmic classes span paraspeckles, nuclear speckles, Cajal bodies, stress granules, P-bodies, neuronal RNA transport granules, U-bodies, and B-bodies, each defined by characteristic composition and behavior. Although each condensate has markers such as *NEAT1* for paraspeckles, SON and SRRM2 for nuclear speckles, coilin for Cajal bodies, G3BP1 and TIA1 for stress granules, Dcp1 and DDX6 for P-bodies, FMRP and Staufen for RNA transport granules, SMN complexes for U-bodies, and Buc for B-bodies, they also share clients and remodeling proteins that enable crosstalk across compartments [7, 10–12, 14, 93–95]. Dissecting component identity and stoichiometry reveals the molecular grammar that sets assembly thresholds, client selectivity, and material states, which in turn govern the rates and fidelity of gene expression reactions. Equally important is understanding how signaling edits condensate formation and dissolution through post-translational modifications, RNA remodeling, and helicase activity, since these controls tune responsiveness and ensure reversibility under stress. The following subsections profile each condensate class by its defining scaffold and marker set, and enumerate constituents, establishing a comparative framework for interpreting function across nuclear and cytoplasmic landscapes.

### Paraspeckles: nuclear condensates for the sequestration and regulation of select RNAs

**Fig. 2:**
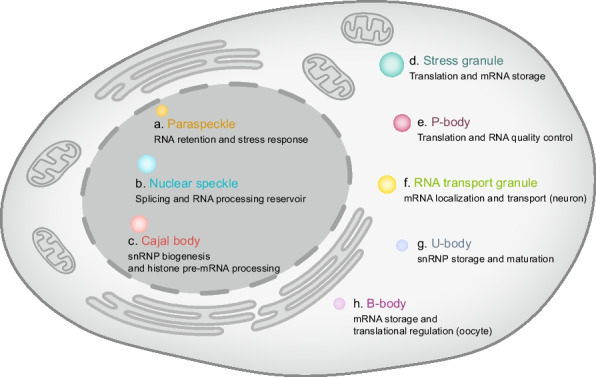
Major classes of biomolecular condensates and their specialized functions. **a** Paraspeckles, organized by *NEAT1* long non-coding RNA, retain hyper-edited or regulatory transcripts and mediate stress-responsive nuclear RNA control. **b** Nuclear speckles act as dynamic reservoirs for splicing factors and RNA-processing machinery, coordinating co-transcriptional pre-mRNA maturation. **c** Cajal bodies concentrate small nuclear ribonucleoprotein (snRNP) biogenesis factors and components of histone gene regulation, thereby supporting spliceosome assembly and transcriptional programs. **d** In the cytoplasm, stress granules form under translational arrest to sequester non-translating messenger ribonucleoprotein complexes (mRNPs), modulate initiation factor availability, and buffer mRNA stability during stress. **e** Processing bodies (P-bodies) serve as hubs for mRNA turnover and translational repression, centralizing decapping, deadenylation, and quality-control pathways. **f** RNA transport granules package selected mRNAs with RNA-binding proteins (RBPs) and ribosomal components to enable long-range trafficking and localized translation in neurons. **g** U-bodies assemble and store snRNPs in the cytoplasm for efficient spliceosomal import. **h** Balbiani bodies (B-bodies) aggregate in quiescent oocytes, incorporating RNAs, proteins, and organelles essential for early development

Paraspeckles are membraneless nuclear ribonucleoprotein (RNP) condensates that assemble on the long noncoding RNA *NEAT1*, particularly the long isoform *NEAT1*_2, and are enriched with core proteins such as SFPQ, FUS, NONO, and PSPC1 (Fig. 2a) [96]. Since their initial description, paraspeckles have become a model for lncRNA-scaffolded nuclear organization and a paradigm for understanding principles of biomolecular condensation in gene regulation. Functionally, they act as buffers and hubs for gene expression. By sequestering proteins such as SFPQ away from chromatin or splice-regulatory sites, paraspeckles modulate transcriptional programs and influence splicing factor availability [97, 98]. They also retain and sort specific RNA classes, including extensively A-to-I–edited transcripts and other RNAs with distinct sequence or structural features, thereby shaping RNA maturation and nuclear export [8, 99]. More broadly, their dynamic formation allows cells to redistribute regulatory capacity within the nucleus as physiological conditions change.

The *NEAT1* locus produces two isoforms: the short, polyadenylated *NEAT1*_1 and the long, RNase P–processed *NEAT1*_2 [100–102]. Among these, *NEAT1*_2 is indispensable for paraspeckle formation, as it provides the architectural scaffold that organizes scores of RBPs through discrete binding domains along its length. The core proteins—SFPQ, FUS, NONO, and PSPC1—assemble into heterodimers and higher-order complexes, while additional components such as DAZAP1, HNRNPH3, HNRNPK, RBM14, and SMARCA4 reinforce structural integrity and expand client recruitment [103–106]. Multivalent interactions involving RNA, RNA-recognition motifs, and IDRs promote phase separation and polymerization-like assembly steps that underpin condensate formation [107, 108].

Paraspeckles are most abundant in differentiated mammalian cells, but are often absent or minimal in embryonic stem cells [8, 109]. However, they can be rapidly induced during differentiation or in response to stress. Notably, *NEAT1* expression is strongly upregulated under diverse stress conditions, including viral infection, hypoxia, oxidative stress, and DNA damage [110–113]. These observations underscore the view that paraspeckles function as stress-responsive condensates, forming to support pro-survival gene-expression states and adaptive RNA processing programs.

### Nuclear speckles: dynamic nuclear reservoirs for pre-mRNA splicing and RNA processing factors

Nuclear speckles are compact ribonucleoprotein condensates in the interchromatin space that concentrate RNA processing factors and coordinate post-transcriptional control (Fig. 2b) [114, 115]. They are enriched in SR family regulators such as SRSF1 and SRSF2 together with the scaffolds SON and SRRM2 [7]. *MALAT1* is a long noncoding RNA that concentrates at speckles and contacts numerous RNA binding proteins [116, 117]. Loss of *MALAT1* generally does not abolish speckle structure while still altering splicing programs in a context-dependent manner [118–120]. Recent work shows that *MALAT1* can modulate alternative splicing through PTBP1 and PSF which provides a route to tune speckle-proximal RNA processing without serving as a structural scaffold [121].

Assembly and growth of nuclear speckles arise from RNA-sensitive, IDR-mediated interactions within the SON–SRRM2 network [122, 123]. Phosphorylation of constitutive proteins plays a crucial role in their subcellular localization [124]. SRSF1 localization is governed by cooperative activities of SRPK1 and CLK1 which sequentially phosphorylate the RS domain to drive nuclear import, speckle incorporation, and mobilization to the nucleoplasm [125, 126]. During mitotic entry broad phosphorylation dissolves speckles and reassembly occurs in early G1 as phosphatase activities rise [114]. Protein phosphatase 1 counteracts kinase-driven dissolution and its increased activity strengthens cohesion enlarges speckles and promotes nuclear retention of polyadenylated RNA [127].

Transcriptional state feeds back on speckle size and composition, since global inhibition of RNA polymerase II enlarges speckles by shifting splicing factors from active genes into the condensate and transcriptional recovery redistributes these clients to chromatin [128, 129]. Genes that localize near speckles display higher spliceosome concentration increased binding to pre-mRNAs and elevated co-transcriptional splicing which links spatial proximity to processing efficiency [130]. APEX-seq and ARTR-seq (reverse transcription–based RBP binding sites sequencing) mapping of nuclear speckle-localized transcriptome reveal accumulation of partially spliced or misprocessed transcripts at speckles, supporting a role in post-transcriptional quality control under stress and signaling cues [131, 132].

### Cajal bodies: maturation of snRNPs and regulation of histone pre-mRNA processing

Cajal bodies are conserved nuclear condensates that concentrate the machinery for small nuclear RNP biogenesis, RNA modification, and specialized RNA processing (Fig. 2c) [133, 134]. The scaffold protein coilin self-associates, binds RNAs and proteins, and sets the interaction landscape for resident factors [135]. Spliceosomal snRNPs transiently accumulate in Cajal bodies together with the SMN complex [134, 136, 137]. It has been reported that the SMN complex assembles the Sm core in the cytoplasm and then imports new snRNPs into the nucleus and traffics to Cajal bodies through the interaction of the coilin RG-box and the SMN Tudor domain. The 3′-end exonuclease TOE1 localizes to Cajal bodies and contributes to the maturation of snRNAs and telomerase RNA [138, 139]. Nopp140 is a largely disordered phosphoprotein that concentrates small Cajal body-specific RNPs (scaRNPs) in Cajal bodies [140]. Depleting Nopp140 releases scaRNPs from Cajal bodies and reduces 2’-O-methylation on snRNAs, which alters compartment residency and affects splicing fidelity [141]. Post-translational modifications program assembly pathways, as global SUMOylation inhibition or SUMO-deficient coilin increases Cajal body number while decreasing size [142].

Cajal bodies collaborate with telomerase pathways and interface with the histone locus body to coordinate RNA processing at gene clusters [143–145]. Their assembly strengthens when the nucleus must process more RNPs and dissolves when transcription wanes [146, 147]. They assemble and enlarge when snRNP supply is perturbed, indicating a role for Cajal bodies in quality control of snRNP assembly [134].

### Stress granules: translational control under stress

Stress granules are among the most extensively studied membraneless organelles formed via phase separation and represent prototypical cytoplasmic RNA–protein condensates that rapidly assemble in response to various cellular stressors (Fig. 2d). These granules are composed of translation initiation factors such as eIF3, eIF4G, and eIF2, together with 40S ribosomal subunits, non-translating mRNAs, and a range of RBPs including G3BP1/2, TIA-1, HuR, and PABP [14, 148–155]. In some cases, signaling proteins are also incorporated into granule structures.

The formation of stress granules is primarily driven by phosphorylation of eIF2α, a process mediated by kinases such as PERK, PKR, GCN2, or HRI in response to stress stimuli [156, 157]. This phosphorylation inhibits translation initiation, leading to polysome disassembly and an increased cytoplasmic concentration of untranslated mRNAs, thereby promoting stress granule nucleation [46, 158, 159]. Scaffold proteins, such as G3BP, function as central organizers of multivalent interaction networks that facilitate phase separation and granule assembly [160]. Specifically, it has been reported that interactions among the three IDRs of G3BP1 regulate phase separation in response to stress, and this is mediated by phosphorylation within the IDRs [46, 161]. G3BP1 induces an RNA-dependent phase separation in response to increased intracellular free RNA concentration, highlighting the influence of its RNA-binding activity and charge distribution on stress granule assembly [162].

Functionally, stress granules are thought to serve as transient storage sites for translationally stalled mRNAs, contributing to global translation suppression and the selective translation of stress-responsive transcripts [163, 164]. These condensates are inherently dynamic and can rapidly disassemble upon stress resolution, thereby allowing the cell to resume normal translational processes. Some evidence suggests that stress granule integrity can influence stress outcomes. Recent reports suggest that normal stress granules are beneficial for cell survival, as impaired stress granule formation accelerates neuronal loss and induces excessive inflammation and immune-mediated apoptosis during viral double-stranded RNA (dsRNA) infection [165, 166]. Additionally, intra-condensate demixing of TDP-43 has been shown to occur within stress granules, suggesting that stress granules may aid in the fluidization of aggregation-prone RBPs [167]. Stress granules are implicated in mRNA triage, determining whether mRNAs are recycled for translation, directed toward degradation via P-bodies or autophagy, or preserved for future use [149, 168]. Emerging evidence suggests that stress granules may also act as signaling hubs, sequestering specific signaling molecules and modulating downstream pathways, such as apoptosis and inflammation.

### P-bodies: cytoplasmic hubs for mRNA turnover

P-bodies are another class of cytoplasmic RNP condensates that play central roles in mRNA decay, mRNA storage, translational repression, and RNA quality control (Fig. 2e) [12, 168–171]. These condensates are enriched in proteins involved in RNA turnover and translational regulation [168, 170]. The core components of P-bodies include the Ccr4-Not deadenylation complex, Lsm1–7 complex, decapping enzymes Dcp1 and Dcp2, and various decapping activators such as Edc3, Pat1, DDX6, and EDC4. In addition, the 5′–3′ exonuclease Xrn1 facilitates mRNA degradation downstream of decapping [170, 172–174]. Beyond this core machinery, P-bodies also incorporate miRNA repression factors such as Argonaute proteins and GW182, translational regulators such as eIF4E, eIF4E-T, and CPEB, and RBPs including Staufen, Rbp1, and TTP, together with components of the nonsense-mediated decay (NMD) pathway such as Upf1p, SMG5, and SMG7 [169, 174–182]. Notably, the presence of certain proteins within P-bodies can vary depending on the type of stress, genetic mutation, or specific cell type [170].

P-body assembly is dynamically modulated by cellular conditions. When translation initiation is globally suppressed, such as during stress, non-translating mRNAs dissociate from ribosomes and accumulate in P-bodies, often leading to increased P-body size [183–185]. Depending on changes in environmental or metabolic states, mRNAs sequestered in P-bodies may be decapped and degraded, or alternatively, exit the P-body and re-enter the translational machinery via polysomes [186]. The fate of each mRNA molecule is determined by a combination of sequence elements, associated proteins, and the prevailing cellular context. Blocking RNA decapping typically increases both the size and number of P-bodies, but macroscopically visible P-bodies are not required for RNA decay [187–191]. Instead, most 5′ → 3′ degradation takes place in soluble cytoplasmic complexes or in small P-bodies that are not readily observed, whereas the large granules primarily sequester RNA. Dcp2 target RNAs are predominantly present in large P-bodies, and decapping with subsequent 5′ → 3′ degradation by Dcp2 and XRN1 likely occurs after these RNAs depart from the large assemblies [188]. This suggests that P-body enrichment by phase separation is crucial for P-body function.

While stress granules sequester translationally repressed mRNAs to protect them from degradation, P-bodies primarily function as mRNA turnover sites. mRNAs encoding enzymes that regulate histone methylation, splicing regulators, proteins involved in RNA poly(A)-deadenylation and capping/decapping, and protein ubiquitination regulators and translation regulators tend to be stored in P-bodies [189, 192]. These reports suggest that P-bodies function as important intracellular mRNA storage sites, potentially affecting chromatin remodeling, transcriptional control, RNA processing, cell division, and differentiation. Although functionally distinct, stress granules and P-bodies are closely interconnected [157, 193, 194]. Under fluctuating stress conditions, the two condensate types can dynamically exchange RNA and protein components and, in some cases, physically interact or transiently fuse, reflecting a high degree of coordination in post-transcriptional gene regulation.

### RNA transport granules: specialized RNP condensates for mRNA storage and localization in neurons

In highly polarized cells such as neurons, the spatial and temporal regulation of gene expression is essential for maintaining cellular architecture, synaptic function, and adaptive responses to external stimuli. Neuronal axons can extend up to one meter in length, and individual synapses may contain between 150 and 20,000 copies of over 1,000–3,000 proteins [195–198]. Maintaining presynaptic protein homeostasis under these conditions is particularly challenging. Neurons overcome these distance-related limitations in part by localizing mRNAs to synaptic compartments and producing proteins on-site through localized translation.

One of the specialized mechanisms that has evolved to facilitate precise spatiotemporal control is the formation of RNA transport granules (Fig. 2f) [199]. These granules are composed of RBPs, ribosomal components, and translationally repressed mRNAs, and function not only as storage units for untranslated transcripts, but also as transport vehicles for long-distance trafficking of mRNA within polarized cells. RNA transport granules dock to other membrane-bound organelles for long-distance transport in mammalian cells, a process known as "hitchhiking". ANXA11, a phosphoinositide-binding protein associated with RNA transport granules, tethers RNA granules to lysosomes [200]. ALS-associated mutations in ANXA11 disrupt the interaction with lysosomes, impairing RNA granule transport. Furthermore, Rab7a endosomes, which harbor RNPs, frequently dock to mitochondria, serving as hotspots for new protein synthesis [201]. Disruption of Rab7a function due to Rab7a mutations significantly reduces axonal protein synthesis, impairs mitochondrial function, and compromises axonal viability. RNA transport granules are known to contain Staufen 2, FMRP, ZBP1, Barentsz, CPEB, TDP-43, DDX6, and DCP1, and some of these components overlap with other RNP condensates, including P-bodies [202–205]. RNA transport granules are not uniform in composition and properties, and they function heterogeneously and dynamically to regulate target mRNAs with high spatiotemporal resolution [205]. In neurons, RNA transport granules are implicated in the delivery and localization of mRNAs to the somatodendritic compartment, thereby enabling fine-tuned localized protein synthesis, which is essential for synaptic plasticity [206–209].

### U-bodies: cytoplasmic condensates for snRNP storage and maturation

snRNPs, which are core components of the spliceosome and are essential for pre-mRNA splicing, function within the nucleus and are enriched in nuclear speckles, Cajal bodies, and histone locus bodies [9, 210–214]. However, the assembly of most snRNPs takes place in the cytoplasm. U-bodies are cytoplasmic condensates that contain snRNPs and critical snRNP assembly factors, and are believed to support the assembly, storage, and regulation of spliceosomal snRNP complexes prior to their nuclear import (Fig. 2g).

U-body-like structures have been observed in Drosophila tissues, as well as in human and Xenopus cells [215]. Notably, U-bodies are consistently associated with P-bodies involved in RNA surveillance and decay. The functional link between U-bodies and P-bodies may involve the regulated release of snRNPs from U bodies in response to RNA turnover rates in P-bodies or may reflect a feedback mechanism whereby snRNP degradation in P-bodies balances their assembly or storage in U bodies, ensuring proper nuclear delivery and homeostasis of snRNP pools.

### B-bodies: evolutionarily conserved cytoplasmic condensates in oocytes

The B-body is a highly conserved, multicomponent cytoplasmic aggregate found in the oocytes of both invertebrates and vertebrates (Fig. 2h) [216–224]. In humans and mice, B-bodies are present in quiescent primary oocytes within primordial follicles and are characterized by their complex composition, which includes not only RNAs and proteins, but also mitochondria, Golgi complexes, and the endoplasmic reticulum (ER) [223–227].

In zebrafish, the formation of B-bodies is driven by microtubule-regulated molecular condensation mediated by the intrinsically disordered protein, Buckyball (Buc), which is essential for B-body assembly [228]. As multiphase condensates, B-bodies play critical roles in RNA storage and translational regulation, contributing to oocyte polarization, germ cell specification, and early embryonic development [216, 219–221, 229, 230].

## Biological functions of biomolecular condensates

Biomolecular condensates are organizing hubs that couple molecular composition to cellular decision making, so defining their functions is essential for explaining how cells coordinate gene expression and adapt to changing conditions. In transcription and splicing regulation, nuclear condensates concentrate core splicing regulators and compartmentalize the transcription apparatus to elevate initiation and elongation [231, 232]. For RNA stability and decay, cytoplasmic condensates scaffold decapping and exonucleolytic pathways, sequester regulons of repressed mRNAs, and thereby set thresholds for surveillance and turnover [192, 233]. During translational reprogramming, cytoplasmic condensates when initiation is limited, redistribute initiation factors and mRNPs, and exhibit stable core substructures that tune the priority of protein synthesis [234]. In the integration of stress signals and regulation of cell survival, the integrated stress response adjusts global translation while cytoplasmic condensates sequester signaling scaffolds such as RACK1 to attenuate apoptosis [75, 235, 236]. Understanding these functions is necessary because functional outputs arise from the interaction grammar and material states of each condensate, which determine client selectivity, reaction rates, and reversibility under stress. Accordingly, the following subsections proceed from transcription and splicing control, to RNA stability and decay, to translational reprogramming, and finally to stress integration and survival, linking for each topic the responsible condensate systems to their defining constituents and operational principles.

### Transcription and splicing regulation

**Fig. 3:**
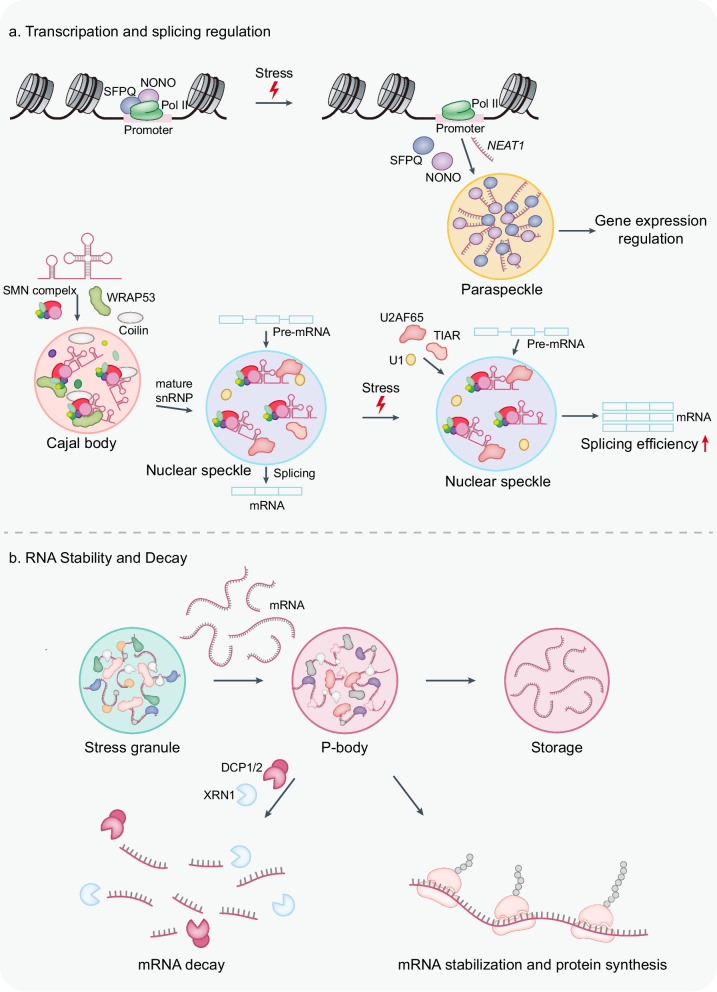
Functional roles of biomolecular condensates in gene expression programs and RNA metabolism. **a** Transcription and splicing regulation. Stress diverts NONO and SFPQ from chromatin into *NEAT1*-scaffolded paraspeckles to modulate transcription. Cajal bodies organize SMN, WRAP53, and coilin to generate snRNPs that supply nuclear speckles. Nuclear speckles act as dynamic reservoirs for spliceosome assembly on nascent pre-mRNAs; under stress, altered recruitment of U2AF65, U1, and TIAR rebalances splice-site usage and can enhance splicing of selected transcripts. **b** RNA stability and decay. Non-translating mRNPs are triaged from stress granules to P-bodies, where DCP1/2 decapping and XRN1-mediated 5′ → 3′ decay drive mRNA turnover, while alternative routing to storage preserves transcripts for later stabilization and translation

Highly transcribed loci are preferentially positioned near nuclear speckles and concentrate coactivators and splicing machinery [237, 238]. Directed tethering of a pre-mRNA to speckle neighborhoods is sufficient to increase splicing efficiency, demonstrating a causal role for speckle proximity in productive gene expression (Fig. 3a) [239]. Under various stress conditions, cells increase the transcription of *NEAT1* and modulate RNA processing to enhance paraspeckle formation, thus making paraspeckles act as a dynamic stress sensor [103]. Paraspeckles influence transcription through sequestration of DNA binding proteins such as SFPQ and NONO away from promoters, which can depress or attenuate transcription at stimulus -responsive genes in a locus-selective manner [240, 241]. The nuclear lamina and speckles are associated with distinct types of retained introns, enriched in genes that function in translation, RNA processing, and the cell cycle, among other processes [132]. Compared with lamina-proximal introns, those retained near speckles are generally shorter and display higher GC content. The distinct signatures at these compartments indicate that speckles and the lamina coordinate control of an extensive set of functionally connected genes essential for gene expression and for multiple steps of the cell cycle.

Cajal bodies serve as assembly sites for spliceosomal snRNPs by concentrating the SMN complex with coilin and WRAP53, which promotes efficient Sm core loading, nuclear import stages, and maturation cycles before release toward transcriptionally active regions [134, 242]. Cajal bodies support splicing indirectly by supplying fully modified and assembled snRNPs to the speckle-rich interchromatin space. Nuclear speckles are enriched in spliceosomal snRNPs and SR-rich regulators, and ensure timely spliceosome supply to nearby transcription units [123, 243]. Ribotoxic stress drives a marked reorganization of nuclear speckles and promotes increased recruitment of splice site recognition factors, including U1, U2AF65, TIAR, and Serine 2 phosphorylated RNA polymerase II [244]. This stress selectively removes retained introns from the pre-mRNA of immediate early genes whose transcription rises during the response. Together, these findings support a model in which nuclear speckles serve as sites of IEG transcription and effective co-transcriptional splicing and as dynamic structures that reconfigure under stress.

### RNA stability and decay

RNA stability is a key determinant of gene expression, particularly under stress conditions, where cells must precisely and rapidly regulate the balance between transcript preservation and degradation. Biomolecular condensates serve as major platforms for post-transcriptional quality control and coordinate RNA surveillance, decay, and storage (Fig. 3b). Unlike nuclear decay systems, these condensates integrate translational signals, decay machinery, and stress-induced regulatory mechanisms to determine RNA fate. Under normal conditions, surveillance pathways, such as NMD, non-stop decay (NSD), and no-go decay (NGD), detect and degrade aberrant transcripts arising from faulty splicing, premature termination codons, ribosome stalling, or structural damage.

P-bodies in the cytoplasm provide scaffolds for selective degradation. These condensates are enriched in degradation enzymes such as DCP1/2 (decapping enzymes), XRN1 (5′–3′ exonuclease), the CCR4–NOT deadenylation complex, Lsm1–7, Pat1, and Edc3, forming dynamic reaction centers [12, 245, 246]. In equilibrium with the cytoplasmic mRNA pool under homeostatic conditions, P-bodies expand in size and number during stress to sequester, degrade, or store translationally silenced mRNAs. Deadenylation plays a central role in mRNA remodeling during P-body formation [247–249]. This remodeling facilitates the recruitment of repressors and decapping complexes, leading to the formation of core P-body mRNPs and subsequent mRNA decay.

In mouse primary oocytes, B-bodies are enriched in TRAL, DDX6, and DCP2 play a dual role in RNA storage and degradation [224]. Additional factors such as DCP1A and mRNACE (mRNA-capping enzyme) are localized to B-bodies, where they may contribute to oocyte arrest by regulating transcript turnover [224, 250]. Notably, B-bodies store approximately 597 mRNAs, including those involved in enzyme binding, cellular component organization, and telomere packaging, as revealed by gene ontology analyses [250]. Their integrity depends on actin and microtubules, and mechanical stress-induced cytoskeletal depolymerization leads to B-body disassembly and activation of primordial follicles.

The demand for RNA quality control increases under stressful conditions. Under conditions such as ER stress or heat shock, defective mRNAs may produce misfolded proteins. Cells prevent this by sequestering these transcripts in stress granules, and if the damage persists, the transcripts are redirected to P-bodies for degradation. This triage system maintains proteostasis by limiting the accumulation of aberrant proteins. RNA helicases such as DDX6 play key roles in unwinding structured or misfolded RNAs and guiding them into decay pathways [251–253]. Cytoplasmic RNA degradation is not stochastic but rather tightly regulated by sequence motifs, chemical modifications, and protein specificity. AU-rich elements regulate stability: HuR stabilizes target mRNAs, whereas AUF1 destabilizes them [254–256]. These proteins often bind to overlapping mRNAs, including p21 and cyclin D1, suggesting competitive or cooperative regulation of transcript fate [257].

Although P-bodies are traditionally considered as degradation centers, not all sequestered RNAs are immediately degraded. Many translationally repressed mRNAs remain intact and serve as regulatory reservoirs that allow rapid translational recovery after stress without the need for de novo transcription. Trafficking between condensates also influences RNA fate; mRNAs may move from stress granules to P-bodies for decay under prolonged stress. U-bodies are functionally linked to P-bodies; for example, the Lsm1–7 complex assembles in U-bodies but is stored in P-bodies, indicating coordinated activity among different condensates based on cell type and stress context [172, 215].

### Translational reprogramming

**Fig. 4:**
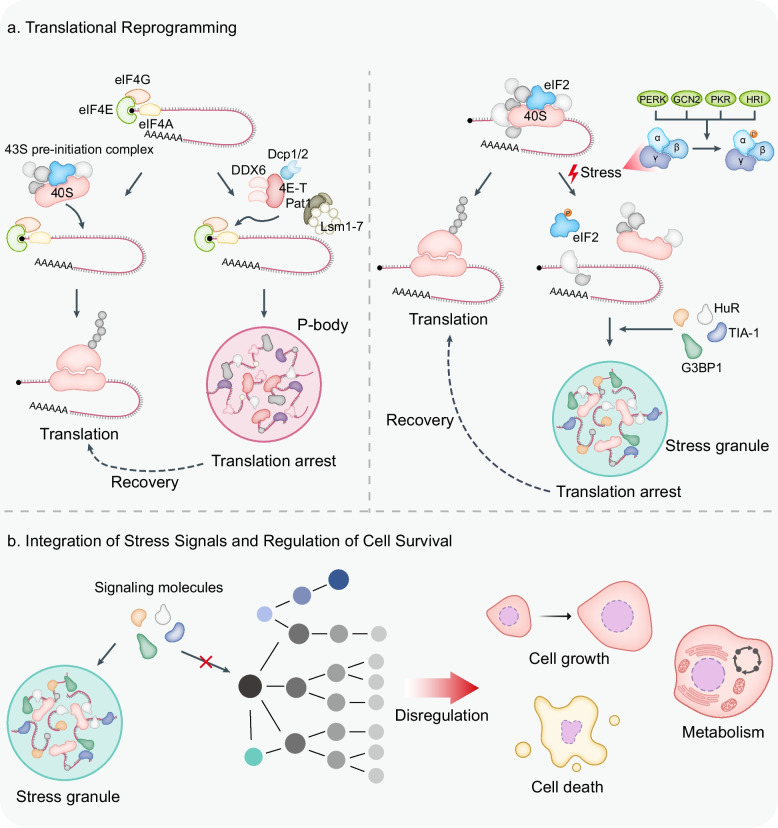
Functional roles of biomolecular condensates in translational regulation, and stress integration. **a** Translational reprogramming. In homeostasis, cap-dependent initiation forms the 43S complex for productive translation. Repressive cues route mRNAs via the DDX6–4E-T–Pat1–Lsm1-7 axis to P-bodies, enforcing arrest. Stress kinases PERK, GCN2, PKR, and HRI phosphorylate eIF2, promoting G3BP1/TIA-1–driven stress-granule assembly that sequesters stalled mRNAs yet permits selective pro-survival synthesis. **b** Integration of stress signals and regulation of cell survival. Stress granules recruit kinases, sequester apoptotic mediators, and regulate signaling for cell survival

Dynamic repression and reprogramming of mRNA translation are central molecular strategies employed by cells to adapt to diverse stressful conditions. Cytoplasmic condensates serve as critical regulatory hubs. Translational regulation via biomolecular condensates extends beyond the mere storage of mRNAs and translation factors and functions as a highly coordinated mechanism for signal integration and dynamic remodeling of the translatome with reversible control (Fig. 4a).

Translation arrest under stress is often initiated by phosphorylation of eIF2α, mediated by stress-sensing kinases such as PERK (ER stress), GCN2 (amino acid deprivation), PKR (viral infection), and HRI (heme depletion, oxidative stress) [258–265]. Phosphorylated eIF2 reduces GDP–GTP exchange on eIF2, reducing the availability of the ternary complex (eIF2–GTP–tRNA_i_^Met^), and thereby globally inhibiting translation initiation. A shortage of ternary complexes results in the accumulation of aberrant 48S pre-initiation complexes (PICs) which cannot recruit the 60S ribosomal subunit and are stalled on mRNAs [155]. Since eIF2α phosphorylation does not impair translation elongation, elongating ribosomes "run off" from mRNAs, leading to polysome disassembly and further accumulation of stalled PICs [266, 267]. These untranslated mRNAs and defective PICs recruit scaffold proteins, such as TIA1 and G3BP1, which are key mechanisms driving the assembly of stress granules. Thus, polysome destabilization promotes the formation of stress granules, whereas polysome stabilization inhibits granule formation.

Importantly, not all mRNAs are equally repressed or sequestered into stress granules. Their fate is determined by multiple factors including regulatory elements in the 5′ and 3′ UTRs, upstream open reading frames (uORFs), RNA modifications like m^6^A, and specific RBP binding profiles. Transcriptomic analyses revealed a broad range of stress granule enrichment levels, ranging from less than 1% to > 95% [268]. Stress granule-associated RNAs are often characterized by long coding regions, extended UTRs, and AU-rich motifs [268, 269]. Although there have been reports of limitations to m^6^A on mRNA enrichment in stress granules, stress granules do not favor long, low-methylation mRNAs and contain highly methylated mRNAs, even if they are short [270, 271]. The apparent presence of long mRNAs in stress granules may be due to long internal exons creating a favorable environment for methylation. mRNAs within stress granules tend to encode genes critical for cell survival and proliferation [268, 269]. While most mRNAs are repressed during stress, those encoding pro-survival proteins may be selectively translated. For example, ATF4, a stress-inducible transcription factor that regulates gene expression during ER stress, amino acid starvation, and oxidative stress, is preferentially translated via a reinitiation mechanism dependent on upstream ORFs in its 5′ UTR under eIF2α phosphorylation [272–276]. Although non-translating mRNAs are preferentially recruited to stress granules and bulk translation initiation is typically suppressed during stress, single-molecule imaging using ATF4 transcripts has shown that stress granule-associated mRNAs can undergo complete translation cycles (initiation, elongation, termination), indicating that translation can occur within stress granules [277]. Therefore, stress granules should be considered as regulators that either inhibit or permit translation depending on their composition, phase state, and stress kinetics. Moreover, m^6^A-modified stress-responsive mRNAs serve as multivalent scaffolds for recruiting YTHDF, facilitating phase separation into P-bodies, stress granules, or neuronal granules [68, 152, 278]. Stress-induced 5′ UTR methylation helps ribosomes distinguish nascent transcripts from pre-existing messages, enabling selective translation. During heat shock, nuclear localization of YTHDF2 protects the methylated 5′ UTRs from demethylation, allowing cap-independent translation of stress-induced transcripts.

P-bodies also repress translation by sequestering translation initiation factors and repressive RBPs, thereby preventing the normal assembly of ribosomal complexes on target mRNAs. Key regulators include DDX6, 4E-T, the Lsm1–7 complex, and Pat1b [279]. The DEAD-box RNA helicase DDX6 impairs the formation of the 43S PIC and blocks eIF4E function, thereby inhibiting ribosome recruitment. It also interacts with DCP1/2 and Edc3 to promote decapping and mRNA degradation. 4E-T binds directly to eIF4E and recruits DDX6 and Pat1b to mRNP complexes, which disrupt eIF4E–eIF4G interactions and inhibit cap-dependent translation [179]. Additionally, P-bodies house miRNA-silenced mRNAs, and remodeling of mRNPs by miRISC or helicases can further repress translation by altering 5′ cap accessibility [175]. Target mRNAs in P-bodies may be decapped and degraded or re-enter translation in response to signaling cues, often through regulated release that requires RBP interaction with the 3′ UTR [280].

In mouse primary oocytes, the RNA metabolism-associated protein TRAL is enriched within B-bodies [224]. Furthermore, MIWI, which interacts with mRNA 5'-caps and piRNAs; GW182, a key mediator of miRNA-induced translational silencing; and AGO2, a central component of the RNA-induced silencing complex, are found both within and outside B-bodies [250, 281–284]. These findings suggest that B-bodies also contribute to RNA-mediated translational repression. While eIF4E is present inside and outside B-bodies, the ribosomal protein S6 required for elongation is absent, indicating low translational activity within the B-body.

Local protein expression requires the translocation of specific mRNAs to specific cellular regions and the regulation of translation within the local pool of mRNAs. For local translation in neurons, RNA transport granules are assembled to transport mRNAs to distal compartments [285]. RNA transport granules contain mRNAs, Staufen proteins, and clusters of ribosomes, but lack eIF4E, eIF4G, and tRNAs, making them incapable of translation [202]. Physiological, developmental, or local stimuli promote depolarization, leading to positional readjustment of RNA transport granules and the subsequent release of mRNAs. Released mRNAs can dynamically shuttle between local mRNPs and polysomes, enabling local protein translation. Therefore, RNA transport granules simultaneously function as a reservoir for translationally repressed mRNAs and release transcripts into an actively translated pool in response to local signals, linking translation and synaptic plasticity.

Reversibility is a key feature of translational repression by biomolecular condensates. Upon stress resolution, phosphatases such as GADD34 and CReP dephosphorylate eIF2α, leading to stress granule disassembly and re-engagement of mRNAs with ribosomes [286, 287]. Stress granules may also be cleared through selective autophagic degradation mediated by the receptor SQSTM1/p62 to restore proteostasis [288–291]. The regulatory mechanisms vary depending on the type of condensate, nature of the stress, and structural features of the mRNA, enabling multilayered translational control that rapidly suppresses global protein synthesis while allowing the selective expression of stress-responsive proteins.

### Integration of stress signals and regulation of cell survival

Biomolecular condensates function not only as passive storage depots but also as active regulatory hubs that interpret and integrate stress signals, directly contributing to cell fate decisions (Fig. 4b). These condensates sense metabolic, oxidative, inflammatory, thermal, or viral stress and, in response, modulate signaling pathways, sequester signaling molecules, and induce the epigenetic reprogramming of gene expression.

Among these, stress granules are the best characterized for their roles in signal modulation. Multiple signaling pathways regulate stress granule formation, and reciprocally, stress granule assembly influences signaling. The mTOR–S6 kinase pathway promotes stress granule assembly [292]. As a central regulator of metabolism, growth, and survival, mTOR signaling is mediated by two complexes, mTORC1 and mTORC2 [293, 294]. The mTORC1 subunit Raptor is recruited to stress granules during stress, sequestering the complex and its downstream kinases, thereby suppressing mTOR signaling to prevent hyperactivation and apoptosis [295–297].

DYRK3 is a dual-specificity kinase that modulates the stability of P-granule-like condensates and mTORC1 signaling under stress [297]. In the inactive state, DYRK3 prevents stress granule dissolution and mTORC1 release through its N-terminal domain. When active, DYRK3 promotes stress granule dissolution and phosphorylates PRAS40, an mTORC1 inhibitor, thereby enhancing mTORC1 signaling [298, 299]. DYRK3 thus connects the condensate dynamics to cell signaling.

AMPK, a master regulator of energy homeostasis, is activated by ATP depletion and influences diverse cellular processes, including growth and autophagy [300–305]. Stress-induced AMPK activation contributes to stress granule assembly, and AMPK localization within stress granules regulates granule size and persistence [306]. AMPK activity is positively correlated with stress granule dynamics and promotes survival under oxidative stress. Stress granules also suppress apoptosis by sequestering death-inducing molecules, such as RACK1 and TRAF2, thereby inhibiting downstream effectors of the p38/JNK pathway under oxidative or ER stress [235, 307]. This compartmentalization prevents the full activation of apoptotic signaling and supports cell survival under adverse conditions.

## Biomolecular condensates in disease

Intracellular biomolecular condensates act as dynamic, spatially confined hubs that orchestrate diverse gene regulatory processes, including the transient storage of translationally stalled mRNAs, transcriptional regulation, RNA processing, stress sensing, and immune response modulation. These condensates possess inherently flexible and dynamic physical properties that allow their compositions and material states to change in response to various cellular conditions and external stimuli.

To maintain homeostasis, cells rely on highly regulated mechanisms that control the formation, maintenance, and dissolution of condensates. The failure of these regulatory systems can result in adverse pathological outcomes. Aberrant phase separation, irreversible solidification of condensates, impaired disassembly, and defective assembly are mechanistic disruptions that have been increasingly recognized in a wide range of diseases, including neurodegenerative disorders, cancer, infections, inflammatory diseases, and rare genetic syndromes. Despite differences in cell type and tissue specificity, dysregulation of condensates commonly leads to the disruption of RNA metabolic pathways, which can trigger cellular dysfunction, chronic inflammation, apoptotic cell death, or aberrant survival signaling.

Importantly, such dysregulation should not be viewed as merely a biochemical anomaly but rather as a central pathogenic driver of disease progression. Elucidating the molecular relationships between the types of condensate abnormalities, associated proteins, pathological mechanisms, and cellular responses in each disease context is essential for understanding the critical roles that these condensates play in pathophysiology. As a result, targeting biomolecular condensates has emerged as a promising strategy for novel therapeutic development, making the investigation of disease-associated remodeling and dysfunction a key focus in current biomedical research.

### Neurodegenerative diseases

Neurodegenerative diseases are characterized by irreversible dysfunction or neuronal loss. Examples include amyotrophic lateral sclerosis (ALS), frontotemporal dementia (FTD), Alzheimer’s disease (AD), and Huntington’s disease (HD). Although these conditions manifest distinct clinical symptoms, such as memory loss, impaired motor coordination, language deficits, and behavioral alterations, they share a common pathophysiological hallmark at the cellular and molecular levels: dysregulation of biomolecular condensates.

**Fig. 5:**
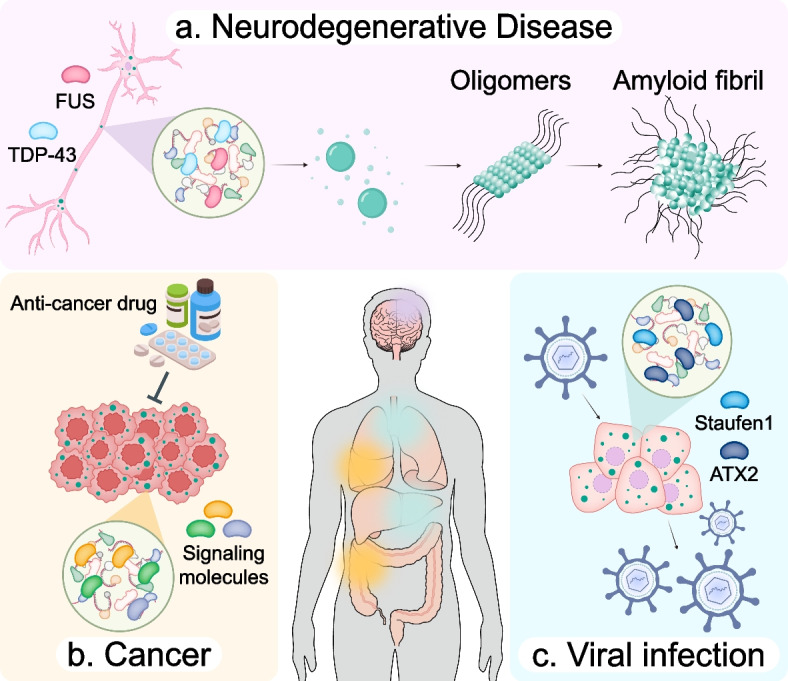
Dysregulation of biomolecular condensates in diverse disease contexts. **a** In stressed neurons, proteins like TDP-43 and FUS mislocalize into solid-like inclusions, disrupting RNA metabolism and protein quality control. **b** Cancer cells amplify biomolecular condensation to reprogram translation, resist apoptosis, and facilitate metastasis. Biomolecular condensates promote cancer therapeutic resistance by sequestering pro-apoptotic factors. **c** Viruses co-opt or disrupt host condensates to facilitate viral RNA replication, evade immune responses, and manipulate host translation

The central RBPs implicated in these diseases are TDP-43 and FUS (Fig. 5a). Both harbor prion-like domains and RNA recognition motifs that confer the ability to undergo phase separation and form RNP condensates. TDP-43 and FUS regulate diverse RNA metabolic processes including transcription, splicing, stability, and transport [308, 309]. Normally localized in the nucleus, TDP-43 shuttles between the nucleus and cytoplasm and accumulates in stress granules under stress conditions, such as oxidative stress, arsenite exposure, or heat shock [310–312]. After stress resolution, TDP-43 typically returns to the nucleus. However, persistent stress leads to prolonged stress granule formation and pathological cytoplasmic aggregation of TDP-43 [313]. TDP-43-related pathologies include mutations in TDP-43, nuclear TDP-43 depletion, cytoplasmic TDP-43 mislocalization, and the formation of insoluble TDP-43 aggregates [314, 315]. HD arises from an expanded CAG repeat within exon 1 of the HTT gene [316, 317]. In both human brain tissues and HD mouse models, TDP-43 shows abnormal distribution, with loss from the nucleus and accumulation of a phosphorylated form in the cytoplasm [318]. TDP-43 function is affected by RNA modifications such as m^6^A and m^1^A, with m^1^A found in CAG-expanded genes [318, 319]. Recently, HD mouse models revealed that nuclear TDP-43 deficiency dysregulates the expression of DNA mismatch repair genes, leading to CAG repeat expansion [320]. Disease-associated mutations, such as A315T, Q343R, and G348C, which are primarily found in ALS, have been shown to promote the formation of larger or aberrant stress granules compared to wild-type TDP-43 [310, 321]. Notably, pathological TDP-43 phosphorylation occurs predominantly near its C-terminal region, with residues such as Ser379, Ser403/404, and Ser409/410 consistently modified in patient samples [322]. Phosphorylated and cytoplasmically mislocalized TDP-43 is detected in 19–57% of AD patients and in up to 50% of FTD cases [323, 324]. Interestingly, cytoplasmic TDP-43 inclusions have been found in more than 97% of the brains of patients with ALS [325]. AD patients with TDP-43 lesions exhibited higher severity of cognitive impairment compared to AD patients without TDP-43 lesions, suggesting that TDP-43 may contribute to neurological dysfunction [326].

FUS behaves similarly, undergoing nucleocytoplasmic shuttling and forming pathological aggregates, in patients with ALS, FTD, and HD [327–332]. Disease-associated mutations in FUS are found in ALS/FTD rather than AD or HD, often affect the C-terminal nuclear localization signals, resulting in impaired nuclear import and enhanced cytoplasmic mislocalization [331, 333–335]. This mislocalization fosters the formation of aberrant stress granules containing mutant FUS. Numerous ALS/FTD-associated mutations in FUS are clustered in IDRs, including the prion-like domain [81, 83, 336]. Arginine mutations reported in ALS alter static binding to RNA, leading to the formation of large condensates, while glycine mutations rapidly lose fluidity and accelerate aging of droplets [337]. Various FUS mutations accelerate the transition from a liquid droplet to a fibrous state, promoting the formation of aggregates observed in patient cells. FUS aggregation is known to deplete functional FUS, disrupting neuronal RNA homeostasis and metabolism, thereby leading to neuronal damage [338]. Nuclear FUS loss can lead to dysfunction in RNA splicing, RNA transport, and DNA damage repair, resulting in dendritic and synaptic defects [339]. Transgenic animal models with FUS knockout or mutations have demonstrated neurodegenerative phenotypes [340–343]. Approximately 10% of ALS cases are inherited, and ALS-associated FUS mutations have been identified in both familial and sporadic ALS cases [328, 344]. While FUS does not display the characteristic pathological changes of clinical AD, abnormal intracellular accumulation of FUS has been observed in 5–10% of clinical FTD cases [338, 345, 346]. Some studies have shown that FUS and TDP-43 interact and colocalize in neuronal inclusion bodies in patients with ALS and FTD, inducing neurodegeneration, suggesting a potential synergistic effect in disease progression [347–349].

Molecular chaperones, including the HSPB8–BAG3–HSP70 complex and valosin-containing protein (VCP), play essential roles in protein quality control (PQC) by preventing the aggregation of misfolded proteins within stress granules and promoting their clearance through disaggregation and autophagy [350–352]. However, the PQC capacity and chaperone expression decline with age and in neurodegenerative diseases, contributing to the pathological solidification of condensates [353]. Additionally, it has recently been discovered that in aged neurons, key splicing proteins are mislocalized and RBPs are downregulated, leading to chronic activation of the RBP-mediated stress response [354]. Aged neurons exhibit impaired overall resilience to acute stress, and depletion of the chaperone HSP90α leads to the formation of chronic stress granules. This suggests that restoring chaperone activity in aged neurons could be a therapeutic approach to prevent neurodegeneration.

*NEAT1*_2 is induced in ALS motor neurons where it scaffolds paraspeckles that recruit TDP-43 and FUS, positioning these disease proteins within nuclear RNA control hubs [355]. *NEAT1* is frequently induced in ALS, and its relationship with nuclear TDP-43 is context dependent. While some ALS motor neurons show an association between loss of nuclear TDP-43 and reduced *NEAT1* with reduced paraspeckle features, other studies report *NEAT1* upregulation in ALS tissue and induction following TDP-43 perturbation in human embryonic stem cell models [356–358]. Increased *NEAT1* expression and paraspeckles were observed in spinal cord tissue from sporadic and familial ALS patients with TDP-43 pathology [357]. *NEAT1* is elevated in human PD and HD postmortem brains, suggesting a disease-related role of *NEAT1*-dependent nuclear structures in patient tissue [359–361].

Tau aggregation in Alzheimer’s disease mislocalizes SRRM2 and other nuclear speckle constituents and perturbs pre-mRNA splicing [362, 363]. Nuclear speckle-specific hnRNP D-like is reduced in aged and Alzheimer mouse hippocampus and acts as a structural speckle component that governs splicing of synapse and cytoskeleton genes required for cognition [364]. Human induced neurons carrying C9ORF72 expansions show that disruption of nuclear speckle integrity globally dysregulates RNA splicing and promotes neuronal toxicity, providing a disease-relevant mechanism for frontotemporal dementia and ALS [365]. Across neurodegenerative diseases, changes in components map directly onto patient pathology, neuronal phenotypes, and mechanistic defects in RNA processing and stress adaptation.

### Cancers

To sustain abnormally high proliferative activity, cancer cells require extensive remodeling of transcription, intracellular RNA metabolism, protein translation, and stress response pathways. This reprogramming often involves the activation of ribosome biogenesis and the repurposing of stress-adaptive systems, enabling tumor cells to survive under hostile microenvironmental conditions such as oxidative stress, nutrient deprivation, hypoxia, and immune infiltration. At the heart of this adaptation is a critical set of membraneless compartments, which are increasingly being recognized as hubs for translational reprogramming and therapeutic resistance (Fig. 5b).

Among these, stress granules represent a prototypical example of biomolecular condensates with strong relevance to tumor biology [366–368]. Persistent or aberrantly enhanced stress granule formation has been linked not only to neurodegeneration but also to tumor initiation and progression in cancers such as colorectal, breast, lung, and prostate cancer [369–372]. In tumor cells, elevated expression of stress granule scaffold proteins, such as G3BP1, G3BP2, and TIA-1, is commonly observed, contributing to robust stress granule assembly in response to cellular stress [373, 374]. Through interactions with signaling cascades, such as MAPK, mTOR, Wnt, and JNK-p53, stress granules serve as platforms that buffer apoptosis and promote cell survival. In colorectal cancer tissues, RBFOX2, a splicing factor that is typically nuclear in normal cells, relocates to cytoplasmic stress granules where it recruits mRNAs encoding cell cycle regulators, including *RB1*, thereby supporting proliferation [375, 376]. In pancreatic cancer, G3BP1 binds to *BART* mRNA, promoting its downregulation, and enhancing tumor cell invasion and metastasis [377, 378]. These examples underscore how stress granule–mediated stress adaptation enhances not only cell proliferation, but also metastatic potential and therapy resistance, correlating with poorer patient prognosis [158, 378–384].

Stress granules also contribute to the development of chemotherapeutic resistance [385]. Bortezomib (Velcade®), a proteasome inhibitor, induces stress granule assembly via HRI-mediated phosphorylation of eIF2α [386]. Knockdown of HRI enhances bortezomib-induced apoptosis, indicating that stress granule formation represents a cellular defense mechanism against chemotherapeutic stress. Cells that failed to form stress granules after bortezomib treatment exhibited heightened susceptibility to apoptosis. Similarly, 5-Fluorouracil (5-FU) induces stress granule formation through PKR activation, resulting in eIF2α phosphorylation and sequestration of pro-apoptotic regulators such as RACK1 [387]. Notably, 5-FU also increases P-body formation, suggesting broad remodeling of RNA granule networks in response to treatment. Lapatinib (Tykerb®), an ERBB2/EGFR inhibitor, activates the PERK–eIF2α pathway, leading to translation suppression and stress granule induction, albeit in a cell type-specific manner [388]. While T47D breast cancer cells showed robust stress granule formation upon lapatinib treatment, MCF-7 cells displayed weaker responses, and MDA-MB-231 and Hs578T cells showed minimal stress granule induction. PERK knockdown sensitizes T47D cells to lapatinib, further implicating PERK-dependent stress granule formation in drug resistance mechanisms. Vinca alkaloids (VAs) such as vinorelbine, vinblastine, and vincristine stimulate stress granule formation by activating PERK, promoting eIF2α phosphorylation, and inhibiting mTOR signaling, which leads to eIF4F complex disassembly via eIF4E–BP1 activation [389]. Blocking VA-induced stress granule formation by inactivating eIF4E–BP1 or inhibiting eIF2α phosphorylation sensitizes tumor cells to apoptosis, suggesting a functional role for stress granules in maintaining cell viability under chemotherapeutic pressure. Moreover, hypoxia-induced stress granule formation in HeLa cells confers resistance to cisplatin and paclitaxel [390]. This resistance can be attenuated by hormonal treatments, such as β-estradiol, progesterone, and stanolone, that disrupt stress granule assembly.

In addition to chemoresistance, stress granules mediate radioresistance. Tumor reoxygenation following radiation promotes stress granule destabilization and HIF-1α upregulation, facilitating adaptive responses that reduce the efficacy of radiotherapy [391]. G3BP1 depletion impairs the ROS-scavenging capacity of cancer cells, diminishes DNA repair efficiency, and increases radiosensitivity [392].

In tumors exposed to hypoxia, DNA damage, nutrient limitation, and cytokines, *NEAT1* transcription rises and paraspeckles enlarge [393, 394]. *NEAT1* is overexpressed in a wide variety of human cancers [395, 396]. Preventing paraspeckle formation by silencing *NEAT1* expression in mice sensitized pre-tumor cells to DNA damage-induced apoptosis and impaired skin tumor formation [394]. *NEAT1* induction under hypoxia also resulted in accelerated cell proliferation, improved clonogenic survival, and reduced apoptosis, all hallmarks of increased tumorigenicity [393]. High tumor *NEAT1* expression in patients with breast cancer is associated with poor survival. Clinical materials show that the *NEAT1*_2 isoform is enriched in aggressive breast tumors with a strong association with HER2 positivity and high grade, linking increased paraspeckle burden to poor clinicopathologic features in patients [397]. Multiple myeloma patient studies and models demonstrate that *NEAT1* induction under hypoxia and nutrient stress promotes survival by enhancing DNA damage response programs and upregulating scaffold proteins within paraspeckles [398]. Oncogenic MUC1-C activates *NEAT1* via NF-κB and MYC and increases expression of core paraspeckle proteins SFPQ, NONO, and FUS [399].

At the molecular level, SRRM2 and SON form a core scaffold that phase separates to build speckle subcompartments and positions highly expressed genes within speckle-associated domains, which can enhance splicing efficiency and promote transcriptional output favored by cancer cells [123]. Cancer progression frequently exploits SR proteins and their kinases, because phosphorylation by SRPK and CLK families controls speckle residency and factor release into spliceosomes, which reprograms isoform choice across cell cycle and migration pathways [400, 401]. A direct link to hematologic malignancy arises from the discovery that SRSF2 reads mRNA 5-methylcytosine (m^5^C) and that the prevalent leukemic mutation at residue P95 impairs m^5^C recognition, alters binding on leukemia-related transcripts, and distorts splicing outputs [402]. CLK2 forms autophosphorylation-regulated condensates that reorganize speckles into enlarged rounded structures while restricting access of splicing factors to pre-mRNA, which demonstrates how tumor-elevated kinase signaling can reshape speckle topology and intron retention programs genome wide [403]. SRPK1 upregulation in clinical cohorts associates with advanced stage and invasion and with oncogenic pathway activation in solid tumors, underscoring that sustained SR protein phosphorylation supports a pro-cancer speckle state at the tissue level [404]. *MALAT1*, a nuclear speckle enriched long noncoding RNA, is elevated across hematologic malignancies and solid tumors and coordinates splicing factor distribution and gene expression programs that favor metastasis, providing a patient-supported axis between speckle lncRNAs and malignancy [405, 406].

Cajal bodies organize snRNP biogenesis and telomerase trafficking through the scaffold protein WRAP53, also called TCAB1, and alterations in this pathway rewire RNA maturation and DNA maintenance processes central to tumor biology [407]. WRAP53 is overexpressed in diverse tumor cell lines and clinical materials, promotes cellular transformation, and protects cancer cells through its roles in Cajal body integrity, SMN complex trafficking, and telomerase localization [408]. High levels of WRAP53 are associated with poor prognosis in head and neck cancer, while reduced WRAP53β expression in epithelial ovarian cancer was associated with impaired DNA damage response and poor patient survival [408, 409].

Through the reprogramming of transcription, translation, RNA processing, and stress response pathways via biomolecular condensate remodeling, cancer cells gain the capacity to evade apoptosis, adapt to hostile environments, and sustain uncontrolled proliferation. In this context, biomolecular condensates act as central regulators of gene expression plasticity in malignancy. Their involvement in cancer progression, metastasis, and therapeutic resistance highlights them as promising biomolecular markers for diagnosis and prognosis as well as potential targets for condensate-directed therapies.

### Virial infection

Infectious diseases caused by RNA and DNA viruses lead to rapid and extensive changes in host gene expression programs and protein synthesis machinery. To replicate their genomes and synthesize viral proteins, viruses co-opt the host's intracellular machinery, including translation initiation complexes, RNA surveillance systems, and RNA–protein condensates, which are among the earliest targets of manipulation (Fig. 5c).

A conserved antiviral mechanism involves *NEAT1* induction that sequesters SFPQ away from promoters so that interleukin-8, DDX60, and RIG-I are transcriptionally de-repressed in infected cells [110, 410]. Clinical materials reveal altered *NEAT1* abundance in COVID-19, with several cohorts reporting elevation in blood from moderate and severe cases and other cohorts reporting decreased serum levels, which underscores disease stage and sampling heterogeneity in patient readouts [411, 412]. Herpes simplex virus 1 produces a striking reconfiguration in which immediate early proteins ICP22 and ICP27 are sufficient to trigger linear and circular splicing of *NEAT1*_2 and thereby remodel the paraspeckle scaffold [413]. Beyond herpesviruses, influenza virus and other RNA viruses induce *NEAT1* and recruit paraspeckle proteins during sensing and interferon cascades, which places this condensate at the center of early innate responses [110]. Paraspeckle factors also affect viral translation control, because PSPC1 has been reported to bind structured viral IRES elements and to antagonize ribosomal engagement [414]. Gammaherpesvirus infection generates virus-induced paraspeckle-like puncta that contain SFPQ, NONO, and hnRNP K together with viral proteins, which demonstrates that DNA viruses can build condensates that mimic core paraspeckle composition during lytic reactivation [415].

Early HIV-1 replication complexes traffic to and accumulate within nuclear speckles in primary macrophages and T cells before reverse transcription is complete, which positions viral cores at speckle-proximal chromatin for subsequent genome integration [416]. HIV-1 infection induces CPSF6 condensates that colocalize with speckle territories, which couples capsid-dependent nuclear routing to speckle-guided chromatin selection [417, 418]. Influenza A virus directs its M segment transcript to nuclear speckles where host factors NS1-BP and hnRNP K cooperate to promote M1 to M2 splicing and license nuclear export, thereby shaping the ratio of ion channel M2 to matrix M1 in infected epithelium [419, 420].

Adenovirus induces Cajal body rearrangements after the onset of viral DNA synthesis and before late gene expression, which temporally links Cajal body remodeling to the transition toward viral structural protein production [421]. The Cajal body protein p80-coilin forms a complex with the adenoviral protein L4-22 K and that coilin facilitates nuclear export of adenoviral mRNAs, while depletion of coilin in A549 cells reduces virus yield and alters nuclear to cytoplasmic mRNA ratios [422]. Large-scale viral protein surveys identified multiple herpesvirus proteins that disrupt Cajal bodies or other nuclear bodies, which indicates that Cajal body dismantling is a recurrent strategy used by nuclear DNA viruses to reshape RNA maturation pathways [423].

Stress granules are integral to the early antiviral defense mechanisms of the host. These membraneless organelles not only act as physical barriers to viral RNA by sequestering it, but also serve as hubs that concentrate innate immune proteins, such as RIG-I, MDA5, RNase L, and OAS, thereby enhancing interferon signaling and antiviral responses [424–426]. Viral infections typically activate PKR, leading to phosphorylation of eIF2α, and the subsequent assembly of stress granules. Although stress granules support host defense by sequestering essential translation initiation factors, many viruses recognize them as obstacles to replication and have evolved diverse strategies to block their formation or promote their disassembly. For instance, the SARS-CoV-2 nucleocapsid protein (N) localizes to stress granules and directly interacts with G3BP1/2, the key stress granule scaffold protein, thereby disrupting their interactions with canonical partners [427, 428]. Furthermore, the N protein preferentially binds to host mRNAs with long 3′UTRs, including *G3BP1* mRNA, modulating their stability and effectively suppressing stress granule assembly as part of a broader suppression of host stress responses. Zika virus activates PKR and the unfolded protein response via NS3 and NS4A proteins, triggering host translational repression [429]. Nonetheless, the expression of viral capsid proteins hijacks key stress granule components such as G3BP1, TIAR, and Caprin-1, suppressing stress granule formation to promote viral replication. Similarly, the capsid proteins of yellow fever virus) and Murray Valley encephalitis virus inhibit stress granule assembly through comparable mechanisms.

Hepatitis C virus (HCV) commandeers G3BP1, ATX2, and PABP1 several key regulators of stress granule dynamics [430]. By 36 h post-infection, P-body formation was disrupted and G3BP1 transiently nucleated stress granules. At 48 h, both DDX6 and G3BP1 exhibited ring-like formations that co-localize with HCV core proteins, indicating late-stage hijacking of P-bodies and stress granule components that are critical for viral RNA replication and translation. Flaviviruses, such as West Nile virus and dengue virus, localize TIA-1/TIAR along with dsRNA and the viral NS3 protein in the perinuclear region, facilitating viral RNA synthesis while simultaneously suppressing stress granules and P-body formation, and thereby preventing translational shutdown in infected cells [431].

The host RBP, Staufen1, which is involved in mRNA decay, localization, and translation, plays a pivotal role in viral replication [432–436]. During HIV-1 infection, Staufen1 interacts directly with the viral GAG protein and is incorporated into the same RNP complex as the viral genomic RNA, forming Staufen1 HIV-1 RNPs (SHRNPs) [437]. These distinct assemblies, which differ from canonical stress granules or P-bodies, are preferentially induced during oxidative stress and interfere with the assembly of antiviral stress granules and P-bodies. Interestingly, Staufen1 depletion enhances SHRNP size, facilitating viral RNA selection, trafficking, and encapsidation.

In some instances, viruses exploit stress granule pathways to delay apoptosis or facilitate survival. For example, reovirus induces eIF2α phosphorylation, which promotes the synthesis of ATF4, a transcription factor involved in stress recovery [438]. Through stress granule formation and the activation of NF-κB, reovirus promotes host cell survival while simultaneously rerouting the translational machinery toward viral mRNAs, thereby enhancing viral replication. Mammalian orthoreovirus transiently induces stress granule formation during the early stages of infection. However, in the later phases, stress granule assembly is markedly reduced, despite elevated levels of phosphorylated eIF2α. During the initial formation of stress granules, numerous viral core particles were localized within the granules. Disassembly of these stress granules permits viral mRNA to evade translational arrest and proceed with efficient translation. Similar transient stress granule induction followed by granule disruption has been observed in poliovirus and Semliki Forest virus infections, highlighting a shared viral strategy for manipulating host RNA stress responses [439, 440].

The interplay between viruses and biomolecular condensates is a critical pathogenic axis in the progression of infectious diseases. Viral exploitation of host condensates such as stress granules and P-bodies enables the precise modulation of antiviral defenses and transcriptional programs. Consequently, RNP condensates act as regulatory hubs that are actively targeted by viruses. Future antiviral strategies may seek to counteract these viral mechanisms by restoring condensate-mediated host defense functions or by selectively inhibiting virus-specific condensate remodeling.

## Biomolecular condensates as therapeutic targets

Therapeutic interest in biomolecular condensates stems from strong evidence that aberrant phase behavior drives pathology across neurodegeneration, cancer, and infection, which makes regulated reprogramming of condensates a tractable route to restore cellular homeostasis. The central concept is to discover condensate-modifying therapeutics that tune assembly thresholds, shift material states, and redirect client partitioning rather than abolish the underlying macromolecules [441, 442].

**Fig. 6:**
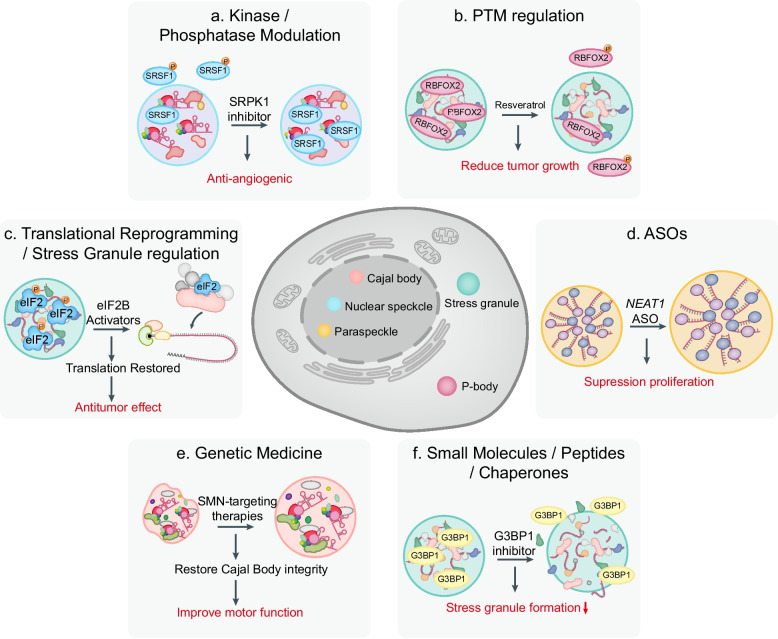
Therapeutic strategies that target biomolecular condensates. **a** Kinase or phosphatase modulation. Inhibition of SRPK1 decreases SRSF1 phosphorylation, alters speckle residency, and suppresses pro-angiogenic splicing.** b** PTM regulation. Small molecules that tune RBP modifications alter condensate partitioning and can restrain tumor growth. **c** Translational reprogramming and stress granule regulation. Modulators of the eIF2–eIF2B axis relieve translational arrest, restore protein synthesis, and produce antitumor effects. **d** Antisense oligonucleotides (ASOs). Depletion of *NEAT1* scaffold RNA disassembles paraspeckles and reduces proliferative signaling. **e** Genetic medicine. SMN-restoring therapies restore Cajal-body integrity, normalize snRNP biogenesis, and improve motor function in motor neuron disease. **f** Small molecules, peptides, and chaperones. Agents that block G3BP1-driven condensation limit stress-granule assembly and relieve translational arrest

Kinase and phosphatase pathways modulate condensate cohesion and client residency, which enables regulation of condensates in vivo and in patients (Fig. 6a). In diabetic rat models, the SRPK1 inhibitor SPHINX31 restored SRSF1 retention in speckles, switched VEGF splicing isoforms toward anti-angiogenic forms, and reduced retinal permeability [443]. The topical SRPK1 inhibitor EXN407 showed safety and signs of biological activity in a Phase 1b or 2a retinal study and is advancing to a Phase 2b program, supporting eye-drop delivery to target speckle-resident SR proteins in diabetic eye disease [444].

A complementary PTM route targets stress granule clients to achieve antitumor effects in vivo (Fig. 6b). Targeted disruption of pathogenic RBP recruitment to stress granules demonstrates anticancer activity [77]. Phospho-valency control can offer a parallel strategy, where kinase nodes remodel material state and translation. For instance, RBFOX2 undergoes PTM-dependent regulation in response to sodium arsenite stress, characterized by increased ubiquitination and reduced phosphorylation, which drives its recruitment to stress granules [375, 376]. Resveratrol treatment restores RBFOX2 phosphorylation and prevents stress granule localization without affecting G3BP1. This selective inhibition reduces RBFOX2 binding to RB1 mRNA and significantly decreases tumor growth and metastasis in mouse melanoma models, highlighting the therapeutic potential of PTM modulation.

Stresses that phosphorylate eIF2α trigger translational arrest and nucleate stress granules around mRNA–ribosome preinitiation complexes (Fig. 6c). In neurodegeneration, prolonged stress granules mature into gel-like assemblies that recruit disease proteins such as TDP-43 and FUS. Direct activation of eIF2B can reverse this cascade, as small-molecule ISR inhibitors, such as ISRIB and 2BAct, promote eIF2B decamer assembly and restore translation [445, 446]. The brain-penetrant eIF2B activator DNL343 prevented and reversed stress granule formation in human iPSC-derived motor neurons carrying TDP-43 mutations and slowed motor decline in a TDP-43 mouse model [447]. In mice with vanishing white matter disease, chronic DNL343 dosing corrected neuronal loss, linking eIF2B activation to disease modification in vivo. Although DNL343 was safe in a Phase 1b study in ALS patients and a Phase 1 study in healthy volunteers, in Phase 2 and Phase 3 studies, DNL343 failed to meet its primary efficacy endpoint and failed to improve key biomarkers, demonstrating the difference between rapid granule reversal and sustained clinical benefit [448]. The DEAD-box helicase eIF4A limits RNA–RNA condensation and suppresses stress granule formation, so catalytic inhibition can remodel translational programs [449]. The clinical eIF4A inhibitor zotatifin reached a recommended Phase 2 dose and showed antitumor activity in estrogen receptor–positive metastatic breast cancer [450, 451].

lncRNAs that scaffold nuclear condensates are druggable with antisense oligonucleotides (ASOs) that deplete targets or rewire isoform choice to remodel condensate mass and composition (Fig. 6d). Isoform-switching ASOs that block the *NEAT1*_1 polyadenylation site raise *NEAT1*_2, expand paraspeckles, and suppress proliferation in neuroblastoma cell models [452]. In ovarian cancer xenograft models, *NEAT1* knockdown suppresses paclitaxel and cisplatin resistance, thereby reducing tumor growth, supporting the potential of *NEAT1* silencing as a chemosensitization strategy [453, 454]. For drug discovery targeting *NEAT1*_2/paraspeckle, a modulator screening toolkit was also developed to identify compounds that increase or decrease *NEAT1*_2 and paraspeckle assembly [455]. Depletion of *MALAT1* using ASOs reduced metastasis and tumor burden in mouse models. In the MMTV-PyMT breast cancer mouse model system, it inhibited lung metastasis and altered the splicing program associated with invasion [456]. Melanoma studies using *MALAT1*-ASO further reported xenograft tumor growth inhibition and MAPK pathway rewiring [457]. In multiple myeloma xenografts, *MALAT1* LNA-gapmeR or nanotube-delivered anti-*MALAT1* oligos trigger apoptosis and impair tumor growth, demonstrating nuclear target engagement and therapeutic effect in vivo [458, 459]. These data establish ASO access to nuclear condensate scaffolds in vivo and justify early-phase trials with pharmacodynamic readouts of paraspeckle or speckle remodeling.

Correcting gene dosage for RNP biogenesis factors can normalize nuclear condensates and reverse downstream pathology in vivo (Fig. 6e). Spinal muscular atrophy arises from SMN deficiency that impairs snRNP biogenesis and disrupts Cajal bodies in motor neurons, linking a defined genetic lesion to condensate dysfunction [460, 461]. The ASO nusinersen enhances SMN2 exon 7 inclusion and, in the SMNΔ7 mouse, restores Cajal body architecture marked by coilin and SMN, normalizes nuclear poly(A) RNA, and improves motor function [462, 463]. Small-molecule risdiplam and AAV9 onasemnogene abeparvovec increase SMN and deliver clinical benefit in SMA [464–466]. SMA therapeutics thus provide a template for condensate restoration through genetic medicine.

Small molecules, peptides, and methyltransferase modulators directly alter protein–protein interfaces and client valency to control stress granule biogenesis and persistence (Fig. 6f). Arginine methylation of RGG motifs tunes phase separation of RBPs, with PRMT1 and PRMT5 constraining condensation of nucleators such as G3BP1 and FUS, whereas demethylation promotes stress granule assembly [467, 468]. The PRMT5 inhibitor GSK3326595 shows signals of clinical activity signals and splicing modulation in Phase 1 and 2 studies in hematologic malignancies [469, 470].

G3BP1 and G3BP2 nucleate many stress granules. Small-molecule screens recently yielded G3Ia and G3Ib that block an interaction surface on G3BP1 and prevent granule formation or promote dissolution [471]. Peptide inhibitors derived from stress granule core proteins or from viral motifs also suppress granule assembly and can reverse sorafenib resistance in cancer cells [472]. Heat-shock chaperones directly remodel condensates. Hsp90 maintains DYRK3 in an active configuration that dissolves stress granules and restarts mTORC1 signaling [473]. Pharmacologic co-inducers of the heat-shock response such as arimoclomol have been evaluated in ALS, though a recent Phase 3 study was negative, highlighting the complexity of translating proteostasis enhancement into clinical benefit [474, 475].

By tuning assembly thresholds, molecular composition, and material states, condensate-directed strategies aim to restore adaptive compartmentalization while limiting disruption of baseline pathways. This perspective enlarges the space of druggable biology because many drivers of cancer, neurodegeneration, and infection operate within condensates or depend on multivalent networks that resist classical site-directed inhibition. Although limitations exist for the clinical application of condensate therapeutics, condensate therapeutics enable precision medicine because genetic variation and cellular context shift phase behavior, creating patient-specific liabilities and predictors of response [27]. Therefore, condensate therapeutics offer a path to transform discovery pipelines and accelerate translation from mechanism to clinic.

## Technological approaches for monitoring biomolecular condensates

Biomolecular condensates can be monitored directly through live-cell visualization or indirectly by tracking the expression and subcellular localization of specific component molecules. One of the most significant technical breakthroughs in the field has been the advent of imaging-based analytical platforms capable of quantitatively and dynamically capturing the formation and dissolution of RNP condensates within live cells. Recent advancements in high- resolution live-cell imaging, including super-resolution microscopy techniques such as stimulated emission depletion (STED) and structured illumination microscopy (SIM), as well as proximity labeling methods, such as APEX, and BioID, and cryo-electron tomography, have transcended the limitations of classical optical resolution [476–481]. These approaches enable the real-time visualization of molecular interactions, mobility, phase transitions, and three-dimensional structural organization within condensates.

Due to the nonlinear and concentration-dependent nature of phase separation, RNP condensates exhibit high spatiotemporal sensitivity in their assembly and disassembly dynamics. Their physical states play a pivotal role in the acute regulation of signal transduction and translation. Thus, technologies enabling real-time manipulation and monitoring of condensate dynamics not only advance disease modeling but also offer a powerful therapeutic platform. In this regard, optogenetics and CRISPR/Cas-based systems are increasingly applied to modulate condensate behavior with a level of precision that exceeds that of conventional drug-based strategies. Currently, these tools are being used to construct real-time surveillance systems capable of detecting the emergence of pathological condensates and tracking the kinetics of condensate formation in situ.

### CRISPR/Cas-based system

Traditionally, many studies have relied on immunostaining of specific proteins as a proxy for condensates [482, 483]. However, this approach has limitations: it is restricted to fixed-cell snapshots, susceptible to localization artifacts, and cannot capture dynamic processes. Although fluorescently tagged protein overexpression systems allow live cell tracking, they may inadvertently induce condensate formation, even under non-stress conditions, particularly when overexpressing certain scaffold proteins. Moreover, stable fluorescent reporter cell lines often require continuous antibiotic selection, which may interfere with endogenous condensate function.

**Fig. 7:**
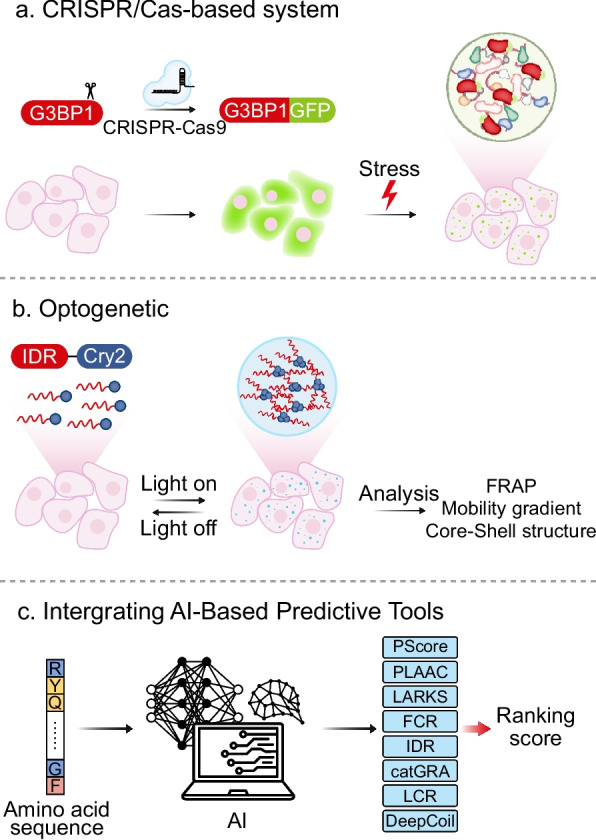
Emerging technologies for real-time monitoring, manipulation, and computational prediction of biomolecular condensates. **a** CRISPR/Cas9-based fluorescent tagging facilitates precise, real-time monitoring of condensate assembly while minimizing artifacts associated with protein overexpression. **b** Optogenetic systems use light-induced protein oligomerization to modulate condensate dynamics at high spatiotemporal resolution, mimicking physiological and diseased states. **c** AI-driven predictive tools integrate phase separation databases and deep-learning methods to predict phase separation tendencies, informing experimental validation and therapeutic strategies

Given that condensates are highly dynamic assemblies governed by reversible phase separations, real-time imaging platforms are essential for accurately analyzing cellular responses and conducting rapid and reliable toxicological assessments. To address the limitations of traditional methods, recent developments have introduced CRISPR/Cas9-mediated endogenous fluorescent protein knock-in systems for the real-time monitoring of condensate dynamics (Fig. 7a). For instance, G3BP1-GFP reporter lines have been successfully established in A549, HEK293, SK-N-SH, and HCT116 cells as well as in lung organoids using CRISPR/Cas9-mediated knock-in strategies [484–486]. These reporter systems faithfully recapitulated endogenous G3BP1 behavior, forming stress granule-like assemblies in response to stress stimuli. Furthermore, they allow the visualization of stress granule formation and disassembly in response to a variety of stressors, including commonly encountered environmental and household chemicals. Such live-cell monitoring systems offer a robust platform for tracking stress granule dynamics in real time and are highly applicable to rapid, high-throughput toxicological screening under various environmental and pathological conditions. Because condensate phase behavior is exquisitely sensitive to small changes in valence or interaction strength, protein tags can, in some cases, perturb condensation [487, 488]. Accordingly, data from tagged constructs should be interpreted cautiously and validated with appropriate in vitro and in vivo controls.

### Optogenetic

Optogenetics has recently emerged as a powerful tool for manipulating protein structure and function within live cells through light-based activation. By enabling reversible and spatiotemporally precise control of biomolecular interactions, optogenetic platforms such as optoDroplets and OptoGranules have provided critical insights into the regulation of phase separation and biomolecular condensate biology (Fig. 7b) [489].

The optoDroplet system utilizes light-induced phase transitions triggered by IDR–Cry2 fusion constructs to study the phase separation dynamics in living cells in real time [4]. This platform allows the precise spatiotemporal induction of condensates formed by the IDRs of key proteins, such as FUS, DDX4, and HNRNPA1. Light activation facilitates their condensation into dynamic liquid droplets that can mature through intermediate gel-like phases into more stable and, in some cases, irreversible aggregates, thereby modeling both physiological and pathological transitions. This system enables selective control of droplet and gel formation kinetics in response to optical stimuli, making it a potent tool for elucidating the molecular underpinnings of both normal and disease-associated phase transitions.

The OptoGranule platform builds upon the optogenetic multimerization of G3BP1 via the photolyase homology region of Cry2, enabling light-inducible assembly of stress granule-like condensates [483]. Light stimulation rapidly drives formation of cytoplasmic granules that recruit hallmark stress granule proteins and RNAs, among them eIF4G, eIF3η, PABP, TDP-43, TIA1, TIAR, FUS, Ataxin-2, GLE1, and polyadenylated RNA. Continued or recurrent induction of OptoGranules leads to toxicity and favors their maturation into neuronal cytoplasmic inclusions that characterize ALS–FTD. These findings demonstrate the utility of optogenetic tools for modeling disease-relevant phase transitions within living cells.

These optogenetic systems offer nontoxic, reversible, and tunable manipulation of condensate formation and can be used to investigate disease mechanisms in models of drug resistance and neurodegeneration. Importantly, they provide high spatiotemporal resolution with minimal off-target effects, rendering them ideal for in vivo control of cellular processes [490, 491]. Future applications may involve cell type-specific implementation, potentially advancing precision medicine strategies targeting dysregulated condensates.

Technological advances have also enabled the experimental reconstruction of phase diagrams, quantitative measurements of mobility gradients, and FRAP to assess the molecular exchange rates and relative concentrations of condensate components. These methodologies have proven to be instrumental in defining the biophysical thresholds and critical functional concentrations required for condensate formation and maintenance.

Increasing efforts are being made to dissect intra-condensate compartmentalization, particularly in the context of stress granules, which are now understood to exhibit a core–shell architecture. In this model, the condensate core is stabilized by scaffold RBPs such as G3BP1 and TIA1, while the outer shell remains highly dynamic, facilitating constant exchange of peripheral proteins and mRNAs with the surrounding cytoplasm [14, 492]. This hierarchical organization challenges the traditional view of condensates as homogeneous entities and supports their characterization as functionally layered and dynamically organized networks. The refinement of such analytical approaches has expanded the field beyond the mere detection of condensates to enable the discrimination between physiological and pathological states based on physical properties and organizational complexity. These insights hold promise for the development of structure-based diagnostic tools and lay the groundwork for a future classification system for “condensopathies”—diseases arising from aberrant RNP condensate behavior.

### Integrating Artificial Intelligence (AI)-based predictive tools

The recent convergence of large-scale databases cataloging proteins with phase separation potential, advances in structural prediction accuracy driven by AlphaFold2, and the growing sophistication of sequence-based interaction modeling have dramatically accelerated the integration of AI and machine learning approaches into biomolecular condensate research (Fig. 7c) [493–498]. In particular, deep-learning-based phase separation prediction models have enabled the identification of phase separation tendencies in specific proteins based solely on sequence data, while simultaneously providing mechanistic insights into condensate dynamics through features such as nucleic acid-binding capacity, IDR composition, and charge or hydrophobicity distribution.

Tools such as PScore quantify phase separation propensity by analyzing planar sp^2^ π–π stacking interactions, which often drive condensate formation through weak aromatic contacts [499]. Prion-like amino acid composition (PLAAC) visualizes and scores protein regions enriched in amino acids characteristic of prion-like domains, thereby highlighting segments that are prone to reversible aggregation [500]. LARKS (Low-complexity aromatic-rich kinked segments) identifies structurally kinked β-sheet–forming motifs that promote phase transition via transient fibrillization of low-complexity regions enriched in aromatic residues [501, 502].

A more recent advancement, catGRANULE 2.0 ROBOT, has significantly expanded its predecessor by integrating structural and sequence-derived data with a comprehensive set of physicochemical descriptors relevant to phase separation [503]. This platform not only predicts phase separation propensity profiles with high resolution but also accurately identifies phase separation-driving regions validated through experimental approaches. Moreover, it allows the prediction of how specific single amino acid variants or disease-associated mutations modulate phase separation behavior, either by enhancing or suppressing condensate formation. Additionally, an in silico design pipeline has also been developed to generate specific, high-affinity binders for IDRs [504]. This method utilizes an induced fit-based binding strategy, combining a physics-based design method with deep learning RF diffusion. This approach offers a general solution to the IDR recognition problem, enabling IDR targeting.

Such computational tools provide a transformative framework for preclinical therapeutic design, enabling the precise modeling of condensate dynamics and their pathological conversion under various genetic and environmental conditions. Ultimately, these technologies pave the way for the development of a bioinformatics-driven condensate atlas, which could be leveraged to predict condensate composition signatures unique to specific disease classes and guide target prioritization in the context of rational therapeutic interventions.

## Conclusion and perspective

Biomolecular condensates, which are formed spontaneously through phase separation between proteins and diverse nucleic acids, have emerged as sophisticated regulatory hubs that enable the spatiotemporal orchestration of gene expression within the cell. Far from being merely sites of molecular accumulation, these membraneless structures dynamically assemble to coordinate key cellular processes, thus playing indispensable roles in maintaining cellular homeostasis and adapting to environmental stress.

Each class of condensates exhibits distinct compositional profiles and biophysical behaviors depending on their molecular constituents and functional contexts. Under physiological conditions, condensates operate as highly reversible and functionally dynamic platforms. However, under pathological stress, genetic mutations, translational disturbances, or energy depletion, they can undergo irreversible transformations such as aberrant solidification or resistance to disassembly. These pathological transitions are recognized as central pathogenic mechanisms in a wide range of disorders, including neurodegenerative diseases, cancer, and infectious diseases. Consequently, condensates have gained increasing attention as biomarkers for disease diagnosis and as therapeutic nodes in the emerging paradigm of precision medicine. Various experimental and therapeutic modalities have been developed to manipulate condensate dynamics in a spatially and temporally controlled manner. These include small-molecule modulators, genetic regulation of protein expression and PTMs, RNA scaffold engineering, optogenetic control systems, and CRISPR-Cas–based precision editing platforms, many of which have demonstrated promising therapeutic efficacy in preclinical and cell-based models [375, 452, 505–508].

Despite rapid progress, the field still lacks a unified quantitative framework that links molecular composition, interaction valency, and cellular context to specific biochemical functions [3, 6]. A central bottleneck is the reliable measurement of material properties in vivo, including viscosity, elasticity, and exchange kinetics, using methods that minimally perturb native networks [82, 509, 510]. Classical FRAP can assess apparent mobility but conflates molecular exchange with network remodeling and is highly sensitive to labeling strategy and expression level [511]. Multiple studies demonstrate that fluorescent protein tags can promote phase separation or reroute aggregation pathways, which implies that legacy datasets require cautious reinterpretation [488, 512]. Solutions to these problems include minimally invasive tagging using small molecule dyes, microscopy protocols that measure cell viscosity and elasticity, and genetically encoded sensors that report saturation concentration and clustering in situ [82, 513–517].

The next question is how to standardize these toolkits across organisms and laboratories so that diffusion, viscosity, and exchange constants measured in one system become predictive in another. Although IDRs, nucleic acid-recognition modules, and multivalent RNA elements coassemble, the stoichiometry, affinity hierarchy, and spatial zoning of clients remain incompletely resolved at endogenous levels [518, 519]. Function requires causality, not only colocalization. Many phenotypes attributed to condensates are still correlations that track changes in abundance under stress or overexpression. The next question is how to engineer perturbations that toggle a condensate’s material state without changing total protein levels, so that function can be assigned to state rather than abundance. Condensates regulate transcription, RNA processing, translation, and signaling, yet most experiments interrogate a single layer, which obscures causal propagation to gene-expression output [1, 520]. Therefore, combining multi-omics and multi-imaging techniques is necessary to link changes in material states to specific changes in gene expression and protein synthesis within the same cell. In addition, future studies should establish a rigorous definition of pathological condensates, quantify disease-specific compositions and functions, and construct diagnostic platforms capable of condensate-based profiling. It is essential to integrate predictive algorithms for therapeutic responsiveness with patient-specific modulation strategies. These goals require a multidisciplinary synthesis of omics-driven analytics, structural biology, cellular biophysics, and artificial intelligence–based modeling.

In conclusion, biomolecular condensates represent not only a physical nexus for the regulation of gene expression and cellular responses, but also a strategic interface for disease detection and therapeutic intervention. The structural principles, functional versatility, pathophysiological relevance, and translational perspectives outlined in this review may provide a conceptual and experimental foundation for realizing condensate-based precision medicine in the years to come.

## Data Availability

Not applicable.
